# Laparoscopic Versus Robot‐Assisted Sacrocolpopexy: A Systematic Review and Meta‐Analysis

**DOI:** 10.1111/1471-0528.70218

**Published:** 2026-03-13

**Authors:** Amerigo Ferrari, Michele Borrelli, Giaele Moretti, Marta Caretto, Elena Pisacreta, Andrea Giannini, Paolo Mannella, Eleonora Russo, Giuseppe Vizzielli, Giuseppe Campagna, Tommaso Simoncini

**Affiliations:** ^1^ Department of Clinical and Experimental Medicine, Division of Obstetrics and Gynaecology University of Pisa Pisa Italy; ^2^ Scuola Superiore Sant'anna, Institute of Management, MeS (Management and Health) Laboratory Pisa Italy; ^3^ Medical Area Department (DAME), Department of Medicine (DMED) University of Udine Udine Italy; ^4^ Clinic of Obstetrics and Gynecology “S. Maria Della Misericordia” University Hospital, Azienda Sanitaria Universitaria Friuli Centrale (ASUFC) Udine Italy; ^5^ Precision Gynecological Surgery Unit, Dipartimento Centro di Eccellenza Donna e Bambino Nascente Fatebenefratelli Gemelli Isola Tiberina Rome Italy

**Keywords:** laparoscopy, meta‐analysis, pelvic organ prolapse, robotic surgery, sacrocolpopexy, systematic review

## Abstract

**Objective:**

To compare the efficacy, safety and perioperative outcomes of robotic‐assisted sacrocolpopexy (RASC) versus laparoscopic sacrocolpopexy (LSC) for the surgical management of apical or multicompartment pelvic organ prolapse (POP).

**Design:**

Systematic review and meta‐analysis of randomised controlled trials (RCTs) and observational studies (PROSPERO CRD42025111099), conducted according to PRISMA guidelines.

**Setting:**

Secondary and tertiary care centres.

**Population:**

Women undergoing sacrocolpopexy for POP.

**Methods:**

Medical databases (PubMed, Scopus, ISI Web of Science, Embase, Cochrane) were searched from inception to April 2025 for studies comparing RASC versus LSC. Risk‐of‐bias was assessed using the Cochrane tool for RCTs and Joanna Briggs Institute checklists for observational studies. Data were meta‐analysed separately by study design using odds ratios (ORs) and standardised mean differences with 95% confidence intervals (CIs), applying fixed‐ or random‐effects models according to heterogeneity.

**Main Outcome Measures:**

Operative time, anatomical outcomes, complications, conversion to laparotomy, recurrence and patient‐reported outcomes.

**Results:**

Five RCTs and twenty‐four observational studies were included. In RCTs, no significant differences between RASC and LSC in operative time, anatomical outcomes or patient‐reported outcomes were found, with similar intraoperative and post‐operative complication rates. In observational studies, perioperative outcomes, complications, mesh exposure, readmissions and recurrence were comparable, but RASC ensured lower conversion to laparotomy (OR 0.2, 95% CI 0.1–0.3).

**Conclusions:**

RASC and LSC provide equivalent anatomical and clinical outcomes with similar morbidity. RASC may be particularly useful in complex or technically demanding cases.

## 1 Introduction

Pelvic organ prolapse (POP) is a common condition whose prevalence is expected to rise substantially with the aging population [1, 2]. Conservative estimates suggest that the number of women affected by symptomatic prolapse will increase by nearly 50% over the next four decades, from approximately 3.3 million to 4.9 million cases [3]. Currently, more than 220 000 women undergo surgical treatment for POP each year, and the estimated reoperation rate approaches 30% [4]. These figures underscore the need for durable, effective surgical procedures that achieve long‐term anatomical restoration while minimising morbidity and cost [5].

Open abdominal sacrocolpopexy (ASC) is regarded as the gold standard for apical and multicompartmental prolapse repair, with reported success rates ranging from 78% to 100% and well‐documented long‐term durability [6, 7]. Compared with vaginal reconstructive approaches, ASC is associated with lower recurrence rates but carries greater perioperative morbidity, including longer operative and recovery times. Consequently, many surgeons continue to favour vaginal approaches in selected patients to limit surgical invasiveness.

The introduction of laparoscopic sacrocolpopexy (LSC) bridged the gap between efficacy and morbidity by maintaining the anatomic success of the open procedure while reducing blood loss, hospital stay and postoperative pain [8]. However, the steep learning curve, the need for advanced laparoscopic suturing skills, and the ergonomic challenges of working in deep pelvic spaces have limited its widespread adoption. To overcome these technical barriers, robotic‐assisted sacrocolpopexy (RASC) was developed [9]. The robotic platform provides improved three‐dimensional visualisation, tremor filtration and wristed instrumentation that enhance dexterity and facilitate dissection and suturing near the sacral promontory. These features may shorten the learning curve and improve surgeon comfort, although their impact on outcomes remains debated [10, 11].

Several comparative studies have evaluated RASC and LSC, yet findings have been inconsistent, particularly regarding operative time and cost‐effectiveness. Earlier meta‐analyses suggested longer operative times for RASC but similar safety and anatomical results [4, 12, 13, 14, 15, 16]. Given the accumulation of new evidence and the evolution of surgical technology, an updated synthesis is needed. The present systematic review and meta‐analysis aim to comprehensively compare robotic‐assisted and laparoscopic sacrocolpopexy, incorporating both randomised controlled trials (RCTs) and observational studies to provide a contemporary evaluation of their relative efficacy and safety.

## 2 Methods

### 2.1 Study Design

This was a systematic review and meta‐analysis. The protocol was developed according to the Preferred Reporting Items for Systematic Reviews and Meta‐Analyses (PRISMA) and registered on PROSPERO (CRD42025111099).

### 2.2 Search Strategy and Selection Criteria

We systematically searched Pubmed, Scopus, ISI Web of Science, Embase and Cochrane from inception to April 2025. Bibliography searching of included studies was also performed to identify relevant literature. The search string was adapted for each database and included the following terms: (robot*) AND (laparoscop*) AND (‘sacrocolpopexy’ OR ‘sacral colpopexy’ OR ‘colposacropexy’). The search was not restricted by study setting or year of publication. Details of the search are outlined in Table S1 of the supporting information.

The review included both experimental and observational studies comparing outcomes associated with robot‐assisted procedures and laparoscopic procedures for sacrocolpopexy. Inclusion criteria were defined to include all studies published in English that included at least five women per arm. Both women who underwent previous hysterectomy and women who had not were included. Outcomes considered for inclusion were grouped into efficiency, clinical, economic and patient‐reported outcomes.

**FIGURE 1:**
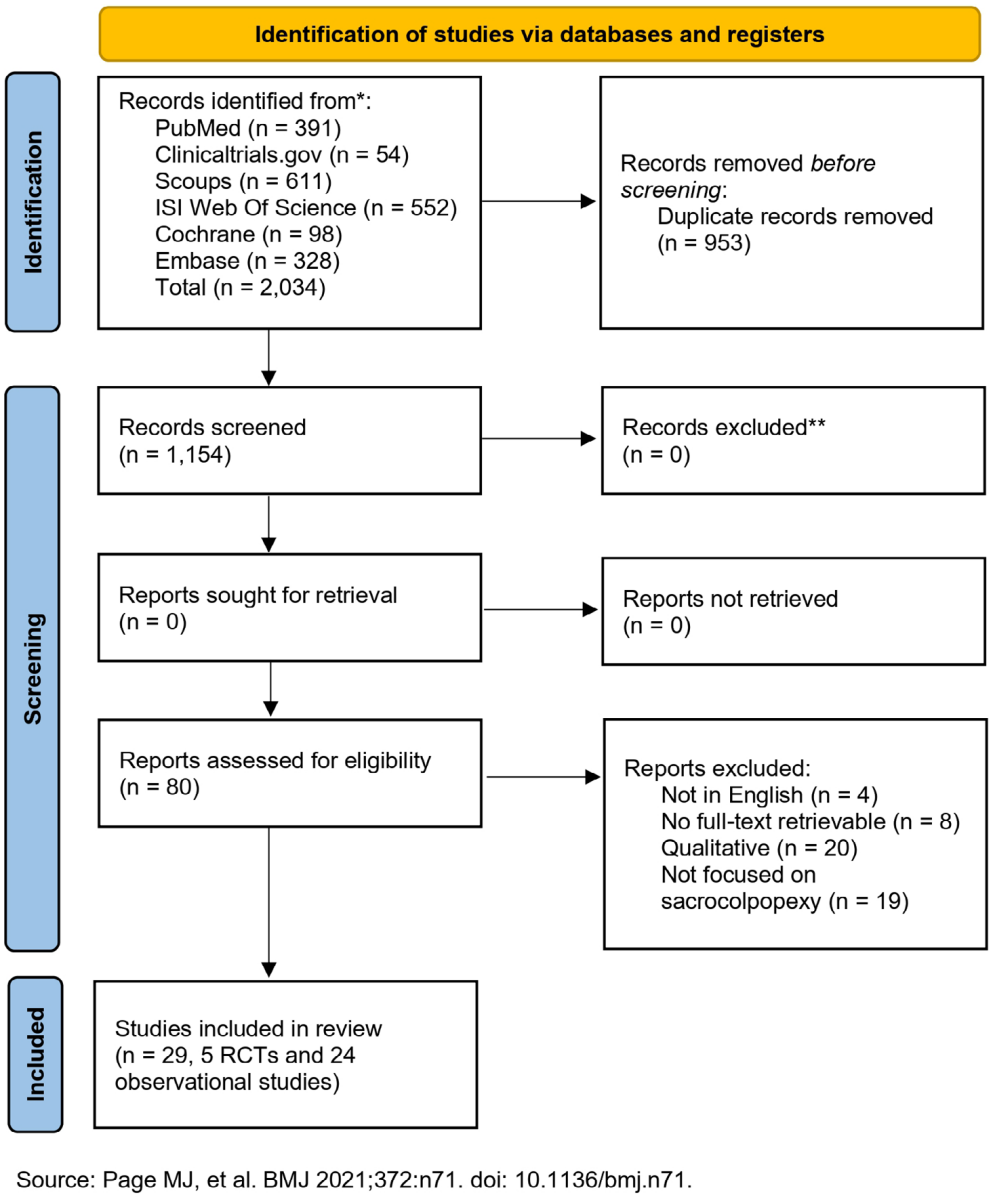
PRISMA flowchart.

After duplicate removals, two authors (AF and MB) underwent a training phase on 20 randomly selected abstracts until a threshold of 80% agreement was reached. Achieving such a level of consensus is recommended by the Cochrane handbook for systematic reviews to ensure a high degree of consistency among reviewers. After the first training phase, the same two authors underwent title/abstract screening of 1025 articles. Disagreements between reviewers were resolved by consensus or by the decision of a third independent reviewer (GM). This was followed by independent full‐text screening and data extraction. The search process is summarised in the PRISMA flowchart reported in Figure 1.

Data extraction was conducted by two reviewers (A.F. and M.B.) using the Microsoft Excel spreadsheet via a standardised data extraction checklist. The extracted information from the studies included, but was not limited to, the following details: author, year of publication, country of study, number of participants per arm, study design, outcome(s) of interest and their definition.

### 2.3 Quality Assessment

Quality assessment of the included studies was independently conducted by three reviewers (A.F., M.B. and G.M.) using the Cochrane Collaboration risk‐of‐bias tool for RCTs and the Joanna Briggs Institute (JBI) Critical Appraisal Checklists for observational studies. As for observational studies, the risk‐of‐bias was classified based on the thresholds reported in previous articles as low risk when at least 70% of the responses were ‘yes’, moderate risk when 50%–60% of the responses were ‘yes’, and high risk when fewer than 50% of the responses were ‘yes [17, 18, 19]’.

### 2.4 Statistical Analysis

A summary table depicting the characteristics of each included study was created to describe the included studies and is reported in Table S2. Two separate meta‐analyses were run for the extracted data from RCTs and observational studies. Data were analysed using STATA 17 and entered in contingency tables.

In conducting the meta‐analysis, we selected appropriate measures of effect size based on the type of outcome reported in the included studies. For continuous outcomes, we calculated standardised mean differences (SMDs) with 95% confidence intervals (CIs). For binary outcomes we used odds ratios (ORs) with corresponding 95% CIs as the measure of association. Both SMDs and ORs were computed for individual studies and then combined to obtain a pooled estimate for each of the selected outcomes, computed as a weighted average of the individual study estimates. We estimated the effects using both fixed and random‐effects models. For RCTs we used either fixed effect models or random‐effects models depending on the levels of heterogeneity. Instead, observational studies were analysed using random‐effect models due to possible differences in study design and reporting that could lead to increased between‐study variance. Studies were also checked for heterogeneity using the *I* ^2^ statistical test, with 75%, 50% and 25% referring to high, moderate and low heterogeneity respectively [20]. In the random effects model, each study was assigned a weight that is reciprocal of its variance, including both within‐study variance and the estimated between‐study variance. Between‐study variance was quantified by computing *τ*_2_ which reflects the extent of heterogeneity across the studies. Publication bias was assessed both visually, using a funnel plot, and statistically, using Egger's test [21]. The model specification was evaluated for each outcome depending on the heterogeneity detected.

## 3 Results

### 3.1 Search Strategy and Study Characteristics

We performed the selection process following the PRISMA guidelines (Figure 1). A total of 1997 articles were retrieved from the databases, and 1025 were selected for title/abstract screening after duplicate removal. Among the 1025 articles, 80 underwent full text screening. By applying the exclusion criteria, five RCTs [22, 23, 24, 25, 26] and twenty‐four observational studies [23, 24, 25, 26, 27, 28, 29, 30, 31, 32, 33, 34, 35, 36, 37, 38, 39, 40, 41, 42, 43, 44, 45, 46] were included in the meta‐analysis.

### 3.2 Quality of the Included Studies

**FIGURE 2:**
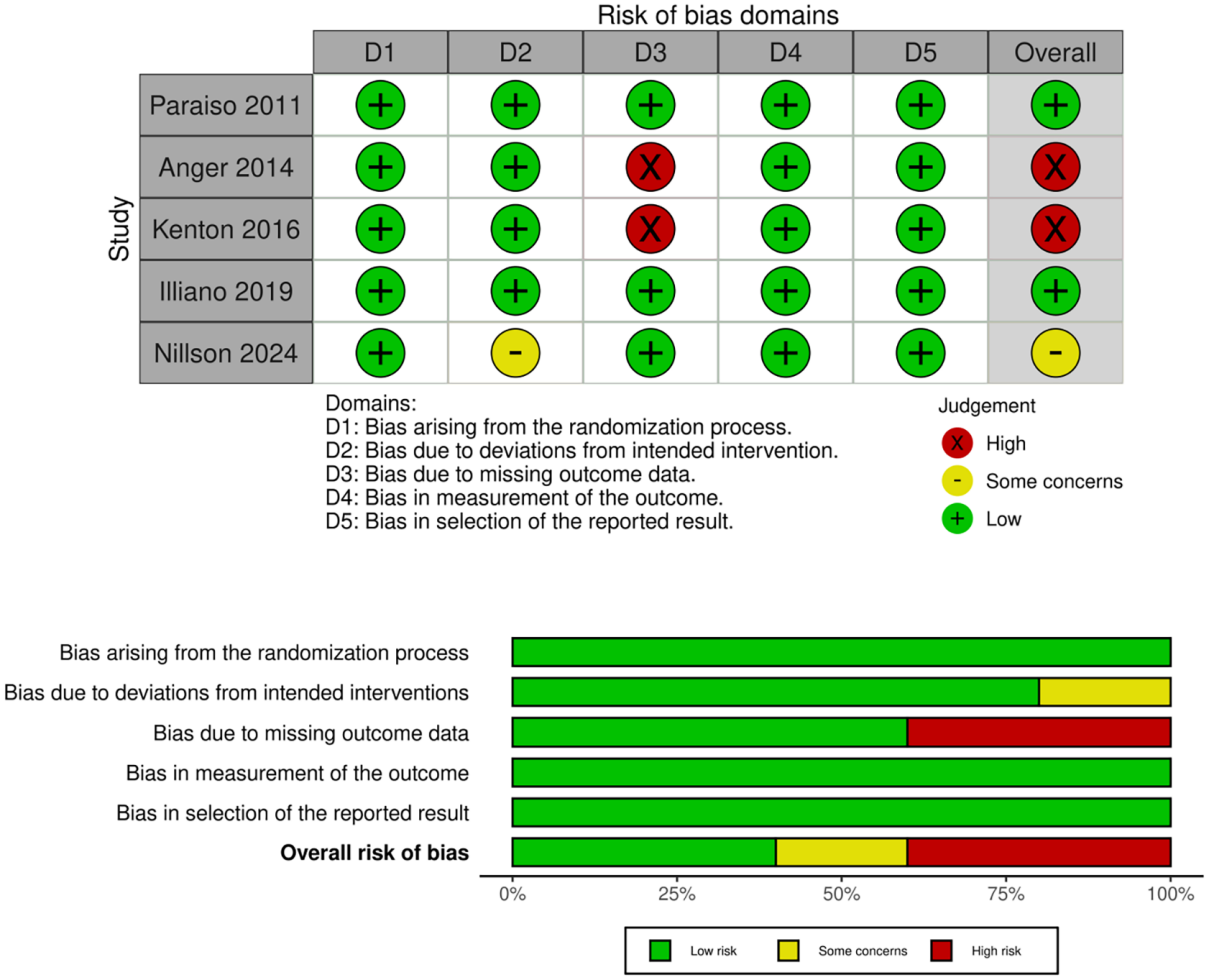
Risk‐of‐bias assessment for RCTs.

Figure 2 reports the results from the risk‐of‐bias assessment for RCTs. The five RCTs showed proper randomization, did not deviate from the intended intervention, and were not biased in the outcome measurement and result selection. Two studies had a high risk‐of‐bias due to missing outcome data. Overall risk‐of‐bias for the RCTs was low for 40% of included studies, moderate for 20% and high for the remaining 40% of studies. As for the 24 observational studies included (Table S3), the risk‐of‐bias was defined as low for 15 papers (62.5%) and moderate for the other 9 papers (37.5%). No study presented a high risk‐of‐bias.

### 3.3 Results of the Meta‐Analysis on RCTs

**FIGURE 3:**
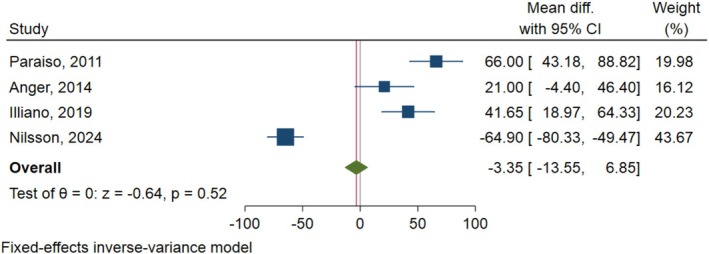
Results of the fixed‐effect meta‐analysis for operative times in RCTs.

Figure 3 reports the results for operative time, showing no statistical difference between RASC and LASC (SMD −3.4, 95% CI −13.6–6.9), although with high levels of heterogeneity (*I* ^2^ 96.51). The exclusion of one study from Nilsson et al. reduced heterogeneity to moderate (*I* ^2^ 70.82), and overall time was 43.4 min longer for RASC compared to LASC (95% CI +18.1–+68.6) (Figure S1).

**FIGURE 4:**
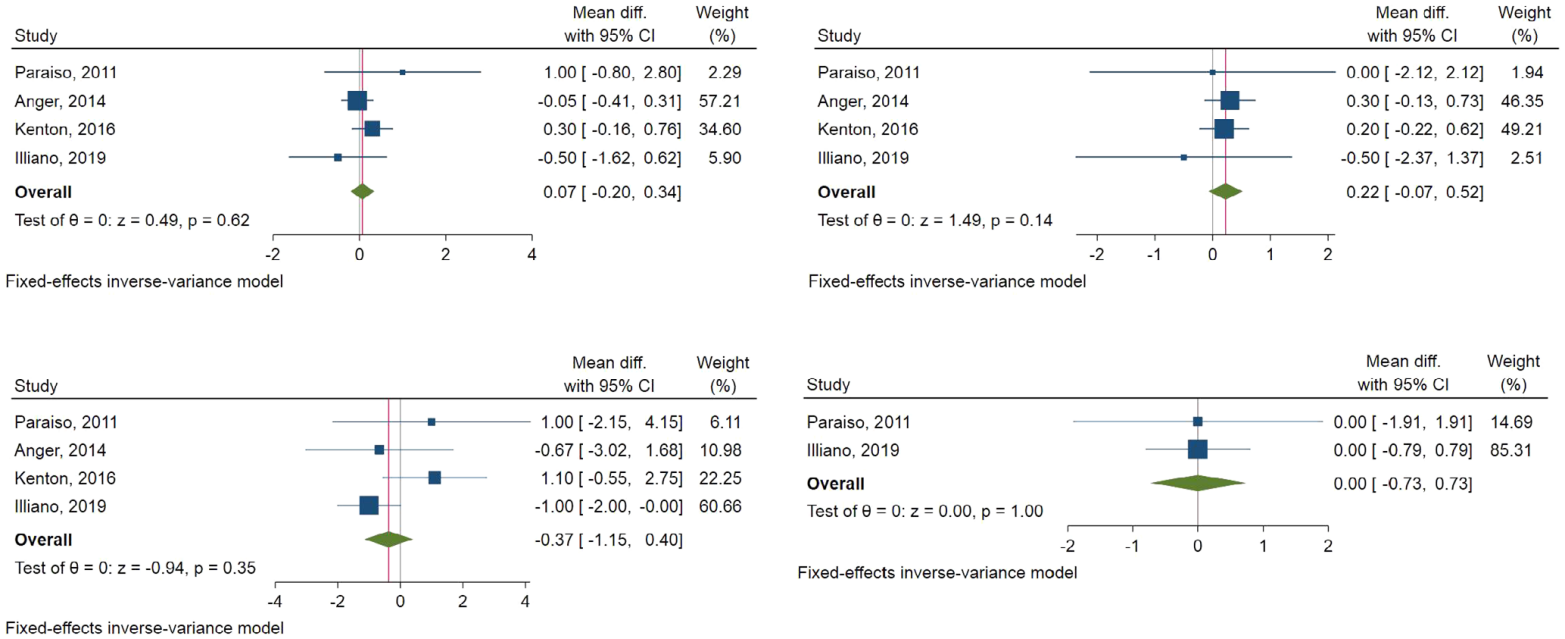
Results of the random‐effect meta‐analysis for anatomical outcomes in RCTs for (a) Point B_a_, (b) Point B_p_, (c) Point C, (d) TVL.

Anatomical outcomes, which are reported in Figure 4, did not differ between the two techniques. As a matter of fact, there was no significant difference in point B_a_ (SMD +0.1, 95% CI −0.2–+0.3), point B_p_ (SMD +0.2, 95% CI −0.1–+0.5), point C (SMD −0.4, 95% CI −1.2–+0.4) and Total Vaginal Length (TVL) (SMD 0.0, 95% CI −0.7–+0.7).

Also patient‐reported outcome measures were not significantly different after RASC vs. LSC (Table 1), as shown by SMDs for UDI (+4.5, 95% CI −5.9–+15.0), POPDI (+9.1, 95% CI −1.6–+19.8), CRADI (+7.4, 95% CI −6.2–21.0), UIQ (−1.4, 95% CI −14.0–+11.2), POPIQ (+6.1, 95% CI −1.6–+13.6) and CRAIQ (−3.9, 95% CI −15.5–+7.6) scores.

**TABLE 1:** Results of the fixed‐effect meta‐analysis for patient‐reported outcomes in RCTs (*N* = number of studies. *I* ^2^ statistic as a measure of heterogeneity).

Patient‐reported outcomes	Robot‐assisted versus laparoscopic mean difference (95% CI)
Urinary Distress Inventory (UDI)	0.07 (−0.20 0.34)
	*N* = 3
	*I* ^2^ = 0.00
Pelvic Organ Prolapse Distress Inventory (POPDI)	0.225 (−0.07 0.52)
	*N* = 3
	*I* ^2^ = 0.00
Colorectal Anal Distress Inventory (CRADI)	−0.374 (−1.15 0.40)
	*N* = 3
	*I* ^2^ = 0.00
Urinary Impact Questionnaire (UIQ)	−1.38 (−14.01 11.24)
	*N* = 3
	*I* ^2^ = 0.00
Pelvic Organ Prolapse Impact Questionnaire (POPIQ)	1.791 (0.22 14.35)
	*N* = 3
	*I* ^2^ = 0.00
Colorectal Anal Distress Impact Questionnaire (CRADIQ)	0.988 (0.23 4.27)
	*N* = 3
	*I* ^2^ = 0.00

Finally, we found no differences in terms of intraoperative (OR 1.0, 95% CI 0.2 to 4.3) and post‐operative (weighted OR 1.8, 95% CI 0.2–14.4) complications, although the latter showed high levels of heterogeneity (*I* ^2^ 86.73) (Figure S2).

### 3.4 Results of the Meta‐Analysis on Observational Studies

Results from the random‐effect meta‐analysis for observational studies are reported in Table 2. We found no significant differences in operating times for RASC versus LSC (SMD +9.0, 95% CI −14.6–+32.5), with high heterogeneity (*I* ^2^ 95.48).

**TABLE 2:** Results of the meta‐analysis for observational studies (*N* = number of studies. *I* ^2^ statistic as a measure of heterogeneity).

Continuous outcomes	Robot‐assisted versus laparoscopic mean difference (95% CI)
Operative time	8.95 (−14.57 32.46)
	*N* = 15
	*I* ^2^ = 95.48
Length of stay	−0.09 (−0.43 0.24)
	*N* = 13
	*I* ^2^ = 76.38
Estimated blood loss	−30.33 (−82.62 21.95)
	*N* = 12
	*I* ^2^ = 98.62
Point B_a_	−0.47 (−0.96 0.01)
	*N* = 4
	*I* ^2^ = 71.97
Point B_p_	−0.004 (−0.34 0.33)
	*N* = 4
	*I* ^2^ = 67.16
Point C	−0.082 (−0.42 0.26)
	*N* = 4
	*I* ^2^ = 0.00

Binary outcomes	Robot‐assisted versus laparoscopic odds ratio (95% CI)
Readmission	0.83 (0.22 3.15)
	*N* = 3
	*I* ^2^ = 3.64
Mesh exposure	0.88 (0.53 1.46)
	*N* = 10
	*I* ^2^ = 0.00
Postoperative complications	0.91 (0.60 1.38)
	*N* = 13
	*I* ^2^ = 38.04
Hernia	1.73 (0.45 6.60)
	*N* = 4
	*I* ^2^ = 0.00
Recurrence	1.48 (0.63 3.52)
	*N* = 7
	*I* ^2^ = 44.97
Perioperative complications	1.225 (0.67 2.25)
	*N* = 6
	*I* ^2^ = 71.44
Intraoperative complications	0.70 (0.38 1.29)
	*N* = 13
	*I* ^2^ = 49.63
Conversion to laparotomy	0.17 (0.11 0.27)
	*N* = 5
	*I* ^2^ = 0.00

Additionally, there was no difference in the average length of stay (SMD −0.1, 95% CI −0.4–+0.2), although with high heterogeneity (*I* ^2^ 76.38). Point B_a_ and Point B_p_ showed both moderate levels of heterogeneity, with the first being slightly lower following RASC than LSC (SMD −0.5, 95% CI −1.0–0.0), whereas no difference was found for point B_p_ (SMD 0.0, 95% CI −0.3–+0.3) and point C (SMD −0.1, 95% CI −0.4–+0.3). Estimated blood loss showed high heterogeneity and was lower for RASC than LSC (SMD −30.3, 95% CI −82.6–22.0), although non‐significantly. There was no significant difference in complication rates for RASC versus LSC, both intraoperatively (OR 0.7, 95% CI 0.4–1.3), perioperatively (OR 1.2, 95% CI 0.7–2.3), and post‐operatively (OR 0.9, 95% CI 0.6–1.4). Heterogeneity was low‐to‐moderate for intraoperative and post‐operative complications, and moderate‐to‐high for perioperative outcomes. We observed no differences in readmission rates (OR 0.8, 95% CI 0.2–3.2) nor in mesh exposure rates (OR 0.9, 95% CI 0.5–1.5). Recurrence was equally likely in both groups (OR 1.5, 95% CI 0.6–3.5). RASC ensured lower chances of conversion to laparotomy (OR 0.2, 95% CI 0.1–0.3) with no heterogeneity detected among included studies, while no difference was found in the frequency of post‐operative hernias (OR 1.7, 95% CI 0.5–6.6). Funnel plots for observational outcomes suggested no substantial publication bias (Figure S3).

## 4 Discussion

### 4.1 Main Findings

In this systematic review and meta‐analysis including both randomised controlled trials and observational studies, we compared robotic‐assisted and laparoscopic sacrocolpopexy for the management of pelvic organ prolapse. A total of 29 studies were included, comprising 5 randomised trials and 24 observational studies. Overall, the methodological quality of RCTs was variable, with most showing low‐to‐moderate risk‐of‐bias and only one study presenting high risk due to missing outcome data. Similarly, most observational studies demonstrated low‐to‐moderate risk‐of‐bias, suggesting an overall acceptable quality of evidence.

The pooled analyses from randomised trials demonstrated no significant differences between robotic and laparoscopic approaches in operative time, anatomical outcomes (points Ba, Bp, C, and total vaginal length) or patient‐reported outcome measures. Similarly, intraoperative and postoperative complication rates were comparable between the two groups.

Findings from observational studies were consistent with those from randomised trials. No significant differences were detected between robotic and laparoscopic sacrocolpopexy in operative time, estimated blood loss, hospital stay or recurrence rates. Both techniques yielded similar anatomical outcomes and comparable rates of intraoperative, perioperative and postoperative complications, as well as mesh exposure and readmissions. However, robotic sacrocolpopexy was associated with a significantly lower rate of conversion to laparotomy.

Our study provides the most updated and comprehensive synthesis of evidence comparing robotic‐assisted and laparoscopic sacrocolpopexy for the management of pelvic organ prolapse. By including both randomised controlled trials and observational studies, it expands the available evidence base beyond previous reviews that had analysed only one type of study design. This methodological approach allows a broader and more realistic assessment of surgical outcomes in both controlled and real‐world clinical contexts.

Earlier meta‐analyses published before 2021 consistently reported longer operative times for the robotic approach compared to laparoscopy, while showing comparable results for blood loss, complication rates and anatomical success. The network meta‐analysis by Chang and colleagues, published in 2022, is the most recent meta‐analysis including only randomised trials and comparing open, laparoscopic and robotic sacrocolpopexy [47]. Their results confirmed significantly longer operative times for RASC versus LSC, with no group differences in terms of estimated blood loss, post‐operative complications or anatomical outcomes. In the previous year, Yang and co‐workers published another meta‐analysis combining both randomised and non‐randomised studies [14]. Although it included a larger number of patients, this study suffered from methodological weaknesses, such as the lack of separate analyses by study design and high heterogeneity across outcomes, which may have reduced the reliability of pooled estimates.

By contrast, our meta‐analysis updates the available evidence with more recent studies and improved methodological rigour. Importantly, the difference in operative times between robotic and laparoscopic sacrocolpopexy, which was significant in earlier analyses, is no longer observed. At the same time, the equivalence in anatomical and clinical outcomes reinforces the notion that both minimally invasive approaches provide safe and effective surgical options for the treatment of pelvic organ prolapse.

### 4.2 Interpretation

The progressive diffusion of robotic technology and the steady increase in surgical experience are likely to further narrow, or even eliminate, the residual differences between robotic‐assisted and laparoscopic sacrocolpopexy. Historically, robotic procedures were associated with longer operative times, largely reflecting the initial learning curve and the technical demands of system setup. However, the loss of significance in operative time differences observed in our meta‐analysis suggests that this gap is closing. Several factors may contribute to this trend, including wider adoption of robotic sacrocolpopexy, increasing surgeon and team experience, incremental improvements in robotic platforms and docking workflows, generational changes in surgical training, and variability in how operative time was defined and reported across studies. Although learning‐curve effects should be attenuated in randomised trials, surgeon experience and case sequence were not consistently reported, so a residual influence of the learning curve cannot be completely excluded. As surgeons and operative teams become increasingly proficient with the robotic platform, and as docking and console times continue to decrease, robotic sacrocolpopexy may soon achieve time efficiency comparable to that of laparoscopy.

Another point is that analyses solely based on length of surgery or complications do not convey the subjective surgical experience of surgeons. Sacrocolpopexy, i.e., suspension of the vaginal apex or cervix to the sacral promontory, is a long and complex procedure involving lengthy anatomical dissection, difficult mesh fixation, and lots of stitching and knot tying. All these steps are reasons for surgeon's exhaustion. A recent multidisciplinary Delphi consensus involving pelvic floor surgeons, experts both in laparoscopic and robotic surgery, highlighted how surgeons with a broad experience in both approaches consider robotic surgery a way to make sacral suspension safer, quicker and more effective [48].

From an economic standpoint, cost has long been a major limiting factor in the widespread adoption of robotic surgery. This scenario, however, is likely to evolve in the near future. The introduction of new robotic systems and the gradual expiration of proprietary patents are fostering market competition and driving down equipment and maintenance costs [49, 50]. Simultaneously, institutional familiarity and increased procedure volumes may improve cost‐effectiveness through optimised resource allocation and reduced intraoperative conversions. Over time, these trends could substantially mitigate the financial gap that currently separates robotic surgery from laparoscopy.

Clinically, the equivalence between the two approaches may lead to a more selective use of robotic surgery, particularly for anatomically complex cases or patients with higher body weight, prior abdominal or pelvic surgery or other factors that increase technical difficulty [51]. In these scenarios, the superior visualisation, precision and ergonomics of the robotic platform can offer tangible intraoperative advantages without compromising safety or efficiency.

In this evolving context, robotic and laparoscopic sacrocolpopexy should be regarded not as competing modalities but as complementary tools within the armamentarium of reconstructive pelvic surgery. As costs decrease and experience grows, robotic surgery might become an equally viable option for complex prolapse repairs in the coming years.

### 4.3 Limitations

Despite the inclusion of both randomised and observational studies, the number of randomised trials remains limited, reducing the statistical power for some comparisons. Some additional perioperative and long‐term outcomes of clinical interest were reported inconsistently across studies and could therefore not be included in the analysis. Moderate‐to‐high heterogeneity was detected across various outcomes, likely reflecting variations in study design, patient selection and surgical expertise. Differences in or lack of the definition of operative time, particularly whether docking and setup were included, may have further contributed to inconsistency in the pooled estimates. Some clinically relevant parameters, such as post‐operative pain, long‐term quality of life and cost analysis, were inconsistently reported and therefore could not be quantitatively assessed. In particular, post‐operative pain, an outcome of specific interest because of potential concerns related to port placement and trocar positioning, was described in too few and too heterogeneous studies to allow a meaningful comparison between approaches. The moderate‐to‐high heterogeneity of complications may be due to their classification into intraoperative, perioperative, and post‐operative complications. Finally, as observational studies inherently carry a higher risk‐of‐bias, residual confounding cannot be entirely excluded. Future well‐designed prospective studies are warranted to confirm these findings and explore long‐term and economic outcomes.

## 5 Conclusion

This systematic review and meta‐analysis, which includes both randomised and observational studies, found no significant differences between robotic‐assisted and laparoscopic sacrocolpopexy in operative time, anatomical outcomes or complication rates. Notably, unlike previous meta‐analyses reporting longer operative times for the robotic approach, our findings indicate comparable procedural durations between techniques. These results suggest that increasing surgical experience and technical refinement may be mitigating previously reported disparities. As costs decrease and accessibility improves, robotic‐assisted sacrocolpopexy could represent an equally valid and potentially preferred option for complex or technically demanding cases in the surgical management of pelvic organ prolapse.

## Author Contributions

A.F., M.B. and G.M. contributed to the conception and design of the work, the acquisition, analysis and interpretation of data, and drafting and reviewing the work. M.C., E.P., A.G., P.M., E.R., G.V. and G.C. contributed to the conception of the work, the interpretation of data, and reviewing the work. T.S. contributed to the conception and design of the work, the interpretation of data, and drafting and reviewing the work. All authors gave the final approval of the version to be published and agreed to be accountable for all aspects of the work.

## Funding

The authors have nothing to report.

## Ethics Statement

Ethical approval was not required for this study as it is based exclusively on publicly available data from previously published research.

## Conflicts of Interest

The authors declare no conflicts of interest.

## Supporting information


**Table S1:** Search strategy for databases.
**Table S2:** Characteristics of studies included in systematic review and meta‐analysis.
**Table S3:** Study Quality Assessment for Observational studies.
**Figure S1:** Results of the random‐effect meta‐analysis for operative times in RCTs with the exclusion of Nilsson et al.
**Figure S2:** Results of the random‐effect meta‐analysis for complications in RCTs.
**Figure S3:**. Funnel plots assessing publication bias for each outcome in observational studies.

## Data Availability

The data that support the findings of this study are available from the corresponding author upon reasonable request.

## References

[1] J. E. Jelovsek , C. Maher , and M. D. Barber , “Pelvic Organ Prolapse,” Lancet 369, no. 9566 (2007): 1027–1038. 17382829 10.1016/S0140-6736(07)60462-0

[2] S. A. Collins , M. O'Shea , N. Dykes , et al., “International Urogynecological Consultation: Clinical Definition of Pelvic Organ Prolapse,” International Urogynecology Journal 32, no. 8 (2021): 2011–2019. 34191102 10.1007/s00192-021-04875-y

[3] J. M. Wu , A. F. Hundley , R. G. Fulton , and E. R. Myers , “Forecasting the Prevalence of Pelvic Floor Disorders in U.S. Women: 2010 to 2050,” Obstetrics and Gynecology 114, no. 6 (2009): 1278–1283. 19935030 10.1097/AOG.0b013e3181c2ce96

[4] C. O. Hudson , G. M. Northington , R. H. Lyles , and D. R. Karp , “Outcomes of Robotic Sacrocolpopexy: A Systematic Review and Meta‐Analysis,” Female Pelvic Medicine & Reconstructive Surgery 20 (2014): 252–260. 25181374 10.1097/SPV.0000000000000070 PMC4374352

[5] A. Ferrari , A. Giannini , C. Seghieri , T. Simoncini , and M. Vainieri , “Regional Practice Variation in Pelvic Organ Prolapse Surgery in Tuscany, Italy: A Retrospective Cohort Study on Administrative Health Data,” BMJ Open 13, no. 3 (2023): e068145. 10.1136/bmjopen-2022-068145 PMC10008403 36882257

[6] P. Mannella , A. Giannini , E. Russo , G. Naldini , and T. Simoncini , “Personalizing Pelvic Floor Reconstructive Surgery in Aging Women,” Maturitas 82, no. 1 (2015): 109–115, 10.1016/j.maturitas.2015.06.032. 26142653

[7] A. Giannini , M. Caretto , E. Russo , P. Mannella , and T. Simoncini , “Advances in Surgical Strategies for Prolapse,” Climacteric 22, no. 1 (2019): 60–64, 10.1080/13697137.2018.1543266. 30721638

[8] A. L. W. M. Coolen , A. M. J. van Oudheusden , B. W. J. Mol , H. W. F. van Eijndhoven , J. P. W. R. Roovers , and M. Y. Bongers , “Laparoscopic Sacrocolpopexy Compared With Open Abdominal Sacrocolpopexy for Vault Prolapse Repair: A Randomised Controlled Trial,” International Urogynecology Journal 28, no. 10 (2017): 1469–1479. 28417153 10.1007/s00192-017-3296-5 PMC5606943

[9] R. K. Lee , A. Mottrie , C. K. Payne , and D. Waltregny , “A Review of the Current Status of Laparoscopic and Robot‐Assisted Sacrocolpopexy for Pelvic Organ Prolapse,” European Urology 65, no. 6 (2014): 1128–1137, 10.1016/j.eururo.2013.12.064. 24433811

[10] H. W. R. Schreuder and R. H. M. Verheijen , “Robotic Surgery,” BJOG: An International Journal of Obstetrics and Gynaecology 116, no. 2 (2009): 198–213. 19076952 10.1111/j.1471-0528.2008.02038.x

[11] J. L. Woelk , E. R. Casiano , A. L. Weaver , B. S. Gostout , E. C. Trabuco , and J. B. Gebhart , “The Learning Curve of Robotic Hysterectomy,” Obstetrics and Gynecology 121, no. 1 (2013): 87–95. 23262932 10.1097/aog.0b013e31827a029e

[12] K. Pan , Y. Zhang , Y. Wang , Y. Wang , and H. Xu , “A Systematic Review and Meta‐Analysis of Conventional Laparoscopic Sacrocolpopexy Versus Robot‐Assisted Laparoscopic Sacrocolpopexy,” International Journal of Gynecology & Obstetrics 132, no. 3 (2016): 284–291, 10.1016/j.ijgo.2015.08.008. 26797199

[13] C. Y. Zhang , Z. J. Sun , J. Yang , T. Xu , L. Zhu , and J. H. Lang , “Sacrocolpopexy Compared With Transvaginal Mesh Surgery: A Systematic Review and Meta‐Analysis,” BJOG: An International Journal of Obstetrics and Gynaecology 128, no. 1 (2021): 14–23. 32426903 10.1111/1471-0528.16324

[14] J. Yang , Y. He , X. Zhang , et al., “Robotic and Laparoscopic Sacrocolpopexy for Pelvic Organ Prolapse: A Systematic Review and Meta‐Analysis,” Annals of Translational Medicine 9, no. 6 (2021): 449. 33850846 10.21037/atm-20-4347 PMC8039662

[15] M. Serati , G. Bogani , P. Sorice , et al., “Robot‐Assisted Sacrocolpopexy for Pelvic Organ Prolapse: A Systematic Review and Meta‐Analysis of Comparative Studies,” European Urology 66, no. 2 (2014): 303–318, 10.1016/j.eururo.2014.02.053. 24631406

[16] L. S. Claydon , B. Whitlow , M. A. Dolcet Artahona , et al., “Robotic Versus Laparoscopic Sacrocolpopexy for Treatment of Prolapse of the Apical Segment of the Vagina: A Systematic Review and Meta‐Analysis,” International Urogynecology Journal 27, no. 3 (2016): 355–366. 26249235 10.1007/s00192-015-2763-0

[17] C. M. Goplen , W. Verbeek , S. H. Kang , et al., “Preoperative Opioid Use Is Associated With Worse Patient Outcomes After Total Joint Arthroplasty: A Systematic Review and Meta‐Analysis,” BMC Musculoskeletal Disorders 20, no. 1 (2019): 234. 31103029 10.1186/s12891-019-2619-8 PMC6525974

[18] A. Elsmore , G. Alayande , E. Mainwaring , et al., “Effects of Race, Ethnicity and Socioeconomic Deprivation on Postpartum Haemorrhage in High‐Income Countries: A Systematic Review and Meta‐Analysis,” BJOG: An International Journal of Obstetrics & Gynaecology 132, no. 13 (2025): 1983–1995, 10.1111/1471-0528.18278. 40631497 PMC12592775

[19] G. Melo , K. L. Dutra , R. Rodrigues Filho , et al., “Association Between Psychotropic Medications and Presence of Sleep Bruxism: A Systematic Review,” Journal of Oral Rehabilitation 45, no. 7 (2018): 545–554, 10.1111/joor.12633. 29663484

[20] E. Demertzidou , C. Chatzakis , P. Cavoretto , et al., “Effect of Mode of Delivery on Perinatal Outcome in Severe Preterm Birth: Systematic Review and Meta‐Analysis,” Ultrasound in Obstetrics & Gynecology 62, no. 4 (2023): 471–485, 10.1002/uog.26241. 37128165

[21] P. C. Zorzato , S. Uccella , G. Biancotto , et al., “Intrauterine Manipulator During Hysterectomy for Endometrial Cancer: A Systematic Review and Meta‐Analysis of Oncologic Outcomes,” American Journal of Obstetrics and Gynecology 230, no. 2 (2024): 185–198, https://linkinghub.elsevier.com/retrieve/pii/S0002937823006105. 37704174 10.1016/j.ajog.2023.09.004

[22] M. F. R. Paraiso , J. E. Jelovsek , A. Frick , C. C. G. Chen , and M. D. Barber , “Laparoscopic Compared With Robotic Sacrocolpopexy for Vaginal Prolapse,” Obstetrics & Gynecology 118, no. 5 (2011): 1005–1013, https://journals.lww.com/00006250‐201111000‐00007. 21979458 10.1097/AOG.0b013e318231537c

[23] E. Illiano , P. Ditonno , K. Giannitsas , G. De Rienzo , V. Bini , and E. Costantini , “Robot‐Assisted vs Laparoscopic Sacrocolpopexy for High‐Stage Pelvic Organ Prolapse: A Prospective, Randomized, Single‐Center Study,” Urology 134 (2019): 116–123, https://linkinghub.elsevier.com/retrieve/pii/S0090429519308362. 31563536 10.1016/j.urology.2019.07.043

[24] W. Nilsson , M. Schmidt , L. Turner , and J. Shepherd , “Comparing Postoperative Pain With Laparoscopic Versus Robotic Sacrocolpopexy,” Journal of Minimally Invasive Gynecology 31, no. 3 (2024): 200–204, https://linkinghub.elsevier.com/retrieve/pii/S1553465023009792. 38013160 10.1016/j.jmig.2023.11.016

[25] J. T. Anger , E. R. Mueller , C. Tarnay , et al., “Robotic Compared With Laparoscopic Sacrocolpopexy,” Obstetrics & Gynecology 123, no. 1 (2014): 5–12, https://journals.lww.com/00006250‐201401000‐00003. 24463657 10.1097/AOG.0000000000000006 PMC4266590

[26] K. Kenton , E. R. Mueller , C. Tarney , C. Bresee , and J. T. Anger , “One‐Year Outcomes After Minimally Invasive Sacrocolpopexy,” Female Pelvic Medicine & Reconstructive Surgery 22, no. 5 (2016): 382–384, https://journals.lww.com/01436319‐201609000‐00020. 27403758 10.1097/SPV.0000000000000300 PMC5070533

[27] M. Patel , D. O'Sullivan , and P. K. Tulikangas , “A Comparison of Costs for Abdominal, Laparoscopic, and Robot‐Assisted Sacral Colpopexy,” International Urogynecology Journal 20, no. 2 (2009): 223–228, http://link.springer.com/10.1007/s00192‐008‐0744‐2. 10.1007/s00192-008-0744-2 18923803

[28] W. M. White , R. K. Goel , M. A. Swartz , C. Moore , R. R. Rackley , and J. H. Kaouk , “Single‐Port Laparoscopic Abdominal Sacral Colpopexy: Initial Experience and Comparative Outcomes,” Urology 74, no. 5 (2009): 1008–1012, https://linkinghub.elsevier.com/retrieve/pii/S0090429509007602. 19716594 10.1016/j.urology.2009.02.086

[29] C. Dehan , S. Marcelle , M. Nisolle , C. Munaut , and L. de Landsheere , “Outcomes of Laparoscopic Versus Robotic‐Assisted Sacrocolpopexy for Pelvic Organ Prolapse–A Comprehensive Retrospective Analysis,” International Urogynecology Journal 35, no. 11 (2024): 2203–2210, https://link.springer.com/10.1007/s00192‐024‐05942‐w. 39432077 10.1007/s00192-024-05942-w

[30] D. D. Antosh , S. A. Grotzke , M. A. McDonald , et al., “Short‐Term Outcomes of Robotic Versus Conventional Laparoscopic Sacral Colpopexy,” Female Pelvic Medicine & Reconstructive Surgery 18, no. 3 (2012): 158–161, https://journals.lww.com/01436319‐201205000‐00006. 22543767 10.1097/SPV.0b013e31824b218d

[31] N. Awad , S. Mustafa , A. Amit , M. Deutsch , J. Eldor‐Itskovitz , and L. Lowenstein , “Implementation of a New Procedure: Laparoscopic Versus Robotic Sacrocolpopexy,” Archives of Gynecology and Obstetrics 287, no. 6 (2013): 1181–1186, http://link.springer.com/10.1007/s00404‐012‐2691‐x. 23274792 10.1007/s00404-012-2691-x

[32] Y. Zhang , X. Jiang , M. Mao , et al., “No Difference in Prolapse Recurrence Rates Between Laparoscopic and Robotic‐Assisted Sacrocolpopexy: A Long‐Term Comparison,” Journal of Minimally Invasive Gynecology 32, no. 5 (2025): 447–453, https://linkinghub.elsevier.com/retrieve/pii/S1553465024015528. 39694439 10.1016/j.jmig.2024.12.006

[33] V. Billone , G. Gullo , G. Perino , et al., “Robotic Versus Mini‐Laparoscopic Colposacropexy to Treat Pelvic Organ Prolapse: A Retrospective Observational Cohort Study and a Medicolegal Perspective,” Journal of Clinical Medicine 13, no. 16 (2024): 4802, https://www.mdpi.com/2077‐0383/13/16/4802. 39200944 10.3390/jcm13164802 PMC11355471

[34] T. N. Thomas , E. R. W. Davidson , E. J. Lampert , M. F. R. Paraiso , and C. A. Ferrando , “Long‐Term Pelvic Organ Prolapse Recurrence and Mesh Exposure Following Sacrocolpopexy,” International Urogynecology Journal 31, no. 9 (2020): 1763–1770, http://link.springer.com/10.1007/s00192‐020‐04291‐8. 32253489 10.1007/s00192-020-04291-8

[35] P. Capmas , E. Suarthana , and M. Larouche , “Conversion Rate of Laparoscopic or Robotic to Open Sacrocolpopexy: Are There Associated Factors and Complications?,” International Urogynecology Journal 32, no. 8 (2021): 2249–2256, https://link.springer.com/10.1007/s00192‐020‐04570‐4. 33104825 10.1007/s00192-020-04570-4

[36] M. Lallemant , C. Tresch , M. Puyraveau , S. Delplanque , M. Cosson , and R. Ramanah , “Evaluating the Morbidity and Long‐Term Efficacy of Laparoscopic Sacrocolpopexy With and Without Robotic Assistance for Pelvic Organ Prolapse,” Journal of Robotic Surgery 15, no. 5 (2021): 785–792, https://link.springer.com/10.1007/s11701‐020‐01177‐1. 33247428 10.1007/s11701-020-01177-1

[37] S. E. Andiman , A. H. Bui , C. Ascher‐Walsh , J. D. Wright , and X. Xu , “Surgical Complications and Hospital Costs in Robot‐Assisted Versus Conventional Laparoscopic Hysterectomy With Concurrent Sacrocolpopexy: Analysis of the Nationwide Readmissions Database,” Female Pelvic Medicine & Reconstructive Surgery 28, no. 5 (2022): e142–e148, https://journals.lww.com/10.1097/SPV.0000000000001133. 35113048 10.1097/SPV.0000000000001133

[38] G. Panico , G. Campagna , L. Vacca , et al., “Minimally Invasive Surgery in Urogynecology: A Comparison of Standard Laparoscopic, Minilaparoscopic, Percutaneous Surgical System, and Robotic Sacral Colpopexy,” Minerva Medica 112, no. 114 (2021): 483–491, https://www.minervamedica.it/index2.php?show=R10Y2021N04A0483. 32272829 10.23736/S0026-4806.20.06561-1

[39] D. Shigemi , A. Okada , and H. Yasunaga , “Postoperative Adverse Events and Re‐Treatment Among Patients Who Have Undergone Laparoscopic and Robotic Sacrocolpopexy for Pelvic Organ Prolapse in Japan,” International Journal of Gynecology & Obstetrics 161, no. 1 (2023): 114–119, https://obgyn.onlinelibrary.wiley.com/doi/10.1002/ijgo.14497. 36200666 10.1002/ijgo.14497

[40] M. Arcieri , A. Morlacco , F. Montebelli , et al., “Sacrocolpopexy After Sub‐Total Hysterectomy vs. Sacral Hysteropexy for Advanced Urogenital Prolapse: A Propensity‐Matched Study,” International Journal of Gynecology & Obstetrics 163, no. 3 (2023): 847–853, https://obgyn.onlinelibrary.wiley.com/doi/10.1002/ijgo.14959. 37382353 10.1002/ijgo.14959

[41] M. Joubert , T. Thubert , J. P. Lefranc , et al., “Comparison of Functional Outcomes With Purely Laparoscopic Sacrocolpopexy and Robot‐Assisted Sacrocolpopexy in Obese Women,” Progrès en Urologie 24, no. 17 (2014): 1106–1113, https://linkinghub.elsevier.com/retrieve/pii/S1166708714005545. 25450756 10.1016/j.purol.2014.09.045

[42] C. A. Unger , M. F. R. Paraiso , J. E. Jelovsek , M. D. Barber , and B. Ridgeway , “Perioperative Adverse Events After Minimally Invasive Abdominal Sacrocolpopexy,” American Journal of Obstetrics and Gynecology 211, no. 5 (2014): 547.e1–547.e8, https://linkinghub.elsevier.com/retrieve/pii/S0002937814008035. 10.1016/j.ajog.2014.07.054 25088866

[43] P. A. Nosti , U. U. Andy , S. Kane , et al., “Outcomes of Abdominal and Minimally Invasive Sacrocolpopexy,” Female Pelvic Medicine & Reconstructive Surgery 20, no. 1 (2014): 33–37, https://journals.lww.com/01436319‐201401000‐00007. 24368486 10.1097/SPV.0000000000000036

[44] G. Cucinella , G. Calagna , G. Romano , et al., “Robotic Versus Laparoscopic Sacrocolpopexy For Apical Prolapse: A Case‐Control Study,” Giornale di Chirurgia—Journal of the Italian Surgical Association 37 (2016): 113–117. 10.11138/gchir/2016.37.3.113 PMC5119697 27734794

[45] M. G. Mueller , K. M. Jacobs , E. R. Mueller , M. G. Abernethy , and K. S. Kenton , “Outcomes in 450 Women After Minimally Invasive Abdominal Sacrocolpopexy for Pelvic Organ Prolapse,” Female Pelvic Medicine & Reconstructive Surgery 22, no. 4 (2016): 267–271, https://journals.lww.com/01436319‐201607000‐00016. 27054799 10.1097/SPV.0000000000000269

[46] N. Evangelopoulos , A. Nessi , and C. Achtari , “Minimally Invasive Sacrocolpopexy: Efficiency of Robotic Assistance Compared to Standard Laparoscopy,” Journal of Robotic Surgery 18, no. 1 (2024): 72, https://link.springer.com/10.1007/s11701‐023‐01799‐1. 38340232 10.1007/s11701-023-01799-1 PMC10858822

[47] C. L. Chang , C. H. Chen , S. S. D. Yang , and S. J. Chang , “An Updated Systematic Review and Network Meta‐Analysis Comparing Open, Laparoscopic and Robotic‐Assisted Sacrocolpopexy for Managing Pelvic Organ Prolapse,” Journal of Robotic Surgery 16, no. 5 (2022): 1037–1045, 10.1007/s11701-021-01329-x. 34779989

[48] T. Simoncini , A. Panattoni , T. Cadenbach‐Blome , et al., “Role of Lateral Suspension for the Treatment of Pelvic Organ Prolapse: A Delphi Survey of Expert Panel,” Surgical Endoscopy 38, no. 8 (2024): 4344–4352, 10.1007/s00464-024-10917-5. 38877319 PMC11289001

[49] C. Innocenzi , M. Criscione , M. Pavone , et al., “The Hugo RAS System in Gynecologic Robotic Surgery: A Systematic Review of Current Applications,” Journal of Robotic Surgery 20, no. 1 (2025): 22, https://link.springer.com/10.1007/s11701‐025‐02973‐3. 41243057 10.1007/s11701-025-02973-3 PMC12620324

[50] T. Hughes , B. Rai , S. Madaan , E. Chedgy , and B. Somani , “The Availability, Cost, Limitations, Learning Curve and Future of Robotic Systems in Urology and Prostate Cancer Surgery,” Journal of Clinical Medicine 12, no. 6 (2023): 2268, https://www.mdpi.com/2077‐0383/12/6/2268. 36983269 10.3390/jcm12062268 PMC10053304

[51] M. A. E. Nobbenhuis , N. Gul , P. Barton‐Smith , O. O'Sullivan , E. Moss , and T. E. J. Ind , “Robotic Surgery in Gynaecology,” BJOG: An International Journal of Obstetrics & Gynaecology 130, no. 1 (2023): e1–e8, https://obgyn.onlinelibrary.wiley.com/doi/10.1111/1471‐0528.17242. 35844092 10.1111/1471-0528.17242

