# Supplementary material for: Laparoscopic Versus Robot‐Assisted Sacrocolpopexy: A Systematic Review and Meta‐Analysis
Source: BJOG. 2026 Mar 13;133(8):1529–38. doi: 10.1111/1471-0528.70218 (PMC13254027; doi:10.1111/1471-0528.70218)

**Laparoscopic versus robot-assisted sacrocolpopexy: a systematic review and meta-analysis**

A. Ferrari ^a, b^, M. Borrelli ^a^, G. Moretti ^b^, M. Caretto ^a^, E. Pisacreta ^a^, A. Giannini ^a^, P. Mannella ^a^, E. Russo ^a^, G. Vizzielli ^c^, G. Campagna ^d^, T. Simoncini ^a^

**SUPPLEMENTARY MATERIAL**

**Table S1**. Search strategy for databases

| **Database** | **Number of articles** |
| --- | --- |
| **Scopus**  (TITLE-ABS-KEY (robot*) AND TITLE-ABS-KEY (laparoscop*) AND TITLE-ABS-KEY ("sacrocolpopex*" OR "sacral W/2 colpopex*" OR "colposacropex*" OR "sacropex*" OR "colpopex*" OR "sacrohysteropex*" OR "hysteropex*")) | 611 |
| **Web Of Science**  TS= (robot* AND laparoscop* AND (sacrocolpopex* OR "sacral NEAR/2 colpopex*" OR colposacropex* OR sacropex* OR colpopex* OR sacrohysteropex* OR hysteropex*)) | 552 |
| **Pubmed**  (robot* [tiab] OR "Robotic Surgical Procedures"[MeSH]) AND (laparoscop* [tiab] OR "Laparoscopy"[MeSH]) AND (sacrocolpopex* [tiab] OR sacral colpopex* [tiab] OR colposacropex* [tiab] OR sacropex* [tiab] OR colpopex* [tiab] OR sacrohysteropex* [tiab] OR hysteropex* [tiab] OR "Sacrocolpopexy"[MeSH]) | 391 |
| **Cochrane**  (robot* AND laparoscop*):ti,ab,kw AND (sacrocolpopex* OR "sacral NEAR/2 colpopex*" OR colposacropex* OR sacropex* OR colpopex* OR sacrohysteropex* OR hysteropex*):ti,ab,kw | 98 |
| **Embase**  robot* AND laparoscop* AND (sacrocolpopex* OR sacral ADJ2 colpopex* OR colposacropex* OR sacropex* OR colpopex* OR sacrohysteropex* OR hysteropex*)  .ti,ab. | 328 |
| **Clinicaltrials.gov** | 54 |

**Table S2.** Characteristics of studies included in systematic review and meta-analysis

*RD= Retrospective design; RCT=Randomized Controlled Trial; N.D=Not Disclosed*

| **Author, Year** | **Country** | **Study Design** | **Purpose/ Objectives** | **Sample Size** | **Outcome(s)** | **Follow up** |
| --- | --- | --- | --- | --- | --- | --- |
| Patel, 2009 | USA | RD | To compare clinical, economic and efficiency outcomes between RASC and LSCP | 10 | Estimated Blood Loss, Length of stay, Total operating time, Costs | N.D. |
| White, 2009 | USA | RD | To compare clinical and efficiency outcomes between RASC and LSCP | 20 | Estimated Blood Loss, Length of stay, Total operating time, Pain | 6 months |
| Chan, 2011 | China | RD | To compare clinical and efficiency outcomes between RASC and LSCP | 36 | Estimated Blood Loss, Length of stay, Total operating time, POP-Q POINT Ba, POP-Q POINT C, POP-Q POINT Bp, TVL, intraoperative complications, postoperative complications | from 16 to 39 |
| Paraiso, 2011 | USA | RCT | To compare clinical and humanistics outcomes between RASC and LSCP | 68 | Total operating time, POP-Q POINT Ba, POP-Q POINT C, POP-Q POINT Bp, TVL, intraoperative complications, postoperative complications, PROMS. | 12 months |
| Tan-Kim, 2011 | USA | RD | To compare clinical, economic and efficiency outcomes between RASC and LSCP | 95 | Estimated Blood Loss, Length of stay, Total operating time, Costs, POP-Q POINT Ba, POP-Q POINT C, POP-Q POINT Bp, mesh erosion, postoperative complications, perioperative complications | 6 months |
| Pulliam, 2012 | USA | RD | To compare clinical and efficiency outcomes between RASC and LSCP | 91 | Estimated Blood Loss, Length of stay, Total operating time, POP-Q POINT Ba, POP-Q POINT C, POP-Q POINT Bp | N.D. |
| Seror, 2012 | France | RD | To compare clinical and efficiency outcomes between RASC and LSCP | 67 | Estimated Blood Loss, Length of stay, Total operating time, mesh erosion, intraoperative complications, postoperative complications | 18 months |
| Antosh, 2012 | USA | RD | To compare clinical and efficiency outcomes between RASC and LSCP | 88 | Estimated Blood Loss, Length of stay, Total operating time, POP-Q POINT Ba, POP-Q POINT C, POP-Q POINT Bp, mesh erosion, intraoperative complications, postoperative complications, readmission | 3 months |
| Anger, 2014 | USA | RCT | To compare clinical, humanistics and efficiency outcomes between RASC and LSCP | 78 | Total operating time, POP-Q POINT Ba, POP-Q POINT C, POP-Q POINT Bp, PROMS | 6 months |
| Awad, 2013 | Israel | RD | To compare clinical and efficiency outcomes between RASC and LSCP | 80 | Estimated Blood Loss, Length of stay, Total operating time | 3 months |
| Joubert, 2014 | France | RD | To compare clinical and efficiency outcomes between RASC and LSCP | 56 | Length of stay, Total operating time, mesh erosion, intraoperative complications, postoperative complications | 12 months |
| Unger, 2014 | USA | RD | To compare clinical outcomes between RASC and LSCP | 370 | Intraoperative complications, mesh erosion, perioperative complications | 6 months |
| Nosti, 2014 | USA | RD | To compare clinical and efficiency outcomes between RASC and LSCP | 525 | Estimated Blood Loss, Length of stay, Total operating time, mesh erosion, intraoperative complications, postoperative complications | 8 months |
| Cucinella, 2016 | Italy | RD | To compare clinical and efficiency outcomes between RASC and LSCP | 40 | Estimated Blood Loss, Length of stay, Total operating time, intraoperative complications, postoperative complications, readmission | 6 months |
| Mueller, 2016 | USA | RD | To compare clinical, efficiency and humanistic outcomes between RASC and LSCP | 458 | Total operating time, Estimated blood loss, Intraoperative complications, Postoperative complications, POP-Q POINT Ba, POP-Q POINT C, POP-Q POINT Bp, PROMS | 3 months |
| Kenton, 2016 | USA | RCT | To compare clinical and humantistics outcomes between RASC and LSCP | 66 | POP-Q POINT Ba, POINT C, POINT Bp, PROMS | 12 months |
| Illiano, 2019 | Italy | RCT | To compare clinical and efficiency outcomes between RASC and LSCP | 100 | Total operating time, POP-Q POINT Ba, POINT C, POINT Bp, TVL, intraoperative complications, postoperative complications | 24 months |
| Thomas, 2020 | USA | RD | To compare clinical outcomes between RASC and LSCP | 526 | Mesh erosion | 6 months |
| Capmas, 2021 | Canada | RD | To compare clinical outcomes between RASC and LSCP | 3295 | Intraoperative complications | N.D. |
| Lallemant, 2021 | France | RD | To compare clinical and efficiency outcomes between RASC and LSCP | 214 | Total operating time, intraoperative complications, postoperative complications, mesh erosion | 32.8 months |
| Özbaşli, 2022 | Turkey | RD | To compare clinical and efficiency outcomes between RASC and LSCP | 68 | Length of stay, intraoperative complications, postoperative complications, readmission | 6 months |
| Andiman, 2022 | USA | RD | To compare clinical and economic outcomes between RASC and LSCP | 5598 | Costs, perioperative complications,  intraoperative complications, postoperative complications | 1 months |
| Arcieri, 2023 | Italy | RD | To compare clinical and efficiency outcomes between RASC and LSCP | 88 | Estimated Blood Loss, Length of stay, intraoperative complications, postoperative complications | 12 months |
| Shigemi, 2023 | Japan | RD | To compare clinical outcomes between RASC and LSCP | 52 | Mesh erosion, intraoperative complications, postoperative complications | 5 months |
| Evangelopoulos, 2024 | Switzerland | RD | To compare clinical and efficiency outcomes between RASC and LSCP | 100 | Postoperative complications, Estimated Blood Loss, Length of stay, Total operating time. | 1.5 months |
| Dehan, 2024 | Belgium | RD | To compare clinical outcomes between RASC and LSCP | 106 | Perioperative complications,  intraoperative complications, postoperative complications | 7 months |
| Billone, 2024 | Italy | RD | To compare clinical and efficiency outcomes between RASC and LSCP | 80 | Estimated Blood Loss, Length of stay, Total operating time, Pain, postoperative complications | 1 months |
| Nilsson, 2024 | USA | RCT | To compare clinical and efficiency outcomes between RASC and LSCP | 90 | Total operating time, intraoperative complications | N.D. |
| Zhang, 2025 | China | RD | To compare clinical and efficiency outcomes between RASC and LSCP | 139 | Estimated Blood Loss, Total operating time, perioperative complications, postoperative complications, mesh erosion | 46 months |

**Table S3.** Study Quality Assessment for Observational studies

| **Author, Year** | **1** | **2** | **3** | **4** | **5** | **6** | **7** | **8** | **9** | **10** | **11** | **RISK** |
| --- | --- | --- | --- | --- | --- | --- | --- | --- | --- | --- | --- | --- |
| Patel, 2009 | N | Y | Y | Y | N | Y | Y | N | N | NA | Y | Moderate |
| White, 2009 | Y | Y | Y | Y | N | Y | Y | Y | N | Y | Y | Low |
| Judd, 2010 | N | Y | Y | Y | Y | Y | Y | Y | Y | NA | Y | Low |
| Chan, 2011 | Y | Y | Y | Y | N | Y | Y | Y | Y | NA | Y | Low |
| Tan-Kim, 2011 | N | Y | Y | Y | N | Y | Y | N | N | NA | Y | Moderate |
| Pulliam, 2012 | Y | Y | Y | Y | N | Y | Y | Y | Y | NA | Y | Low |
| Seror, 2012 | Y | Y | Y | Y | N | Y | Y | Y | Y | NA | Y | Low |
| Antosh, 2012 | Y | Y | Y | Y | N | Y | Y | N | Y | NA | Y | Low |
| Awad, 2013 | Y | Y | Y | Y | N | Y | Y | N | Y | NA | Y | Low |
| Joubert, 2014 | N | Y | Y | Y | N | Y | Y | Y | Y | NA | Y | Low |
| Unger, 2014 | N | Y | Y | Y | N | Y | Y | N | Y | NA | Y | Moderate |
| Nosti, 2014 | N | Y | Y | Y | Y | Y | Y | Y | N | N | Y | Low |
| Cucinella, 2016 | Y | Y | Y | N | NA | Y | Y | Y | Y | NA | Y | Low |
| Thomas, 2020 | N | Y | Y | Y | N | Y | Y | N | N | N | Y | Moderate |
| Capmas, 2021 | Y | Y | Y | Y | Y | Y | Y | N | Y | NA | Y | Low |
| Lallemant, 2021 | Y | Y | Y | Y | Y | Y | Y | Y | Y | NA | Y | Low |
| Özbaşli, 2022 | N | Y | Y | Y | N | Y | Y | N | Y | NA | Y | Moderate |
| Andiman, 2022 | Y | Y | Y | Y | Y | Y | Y | N | Y | NA | Y | Low |
| Arcieri, 2023 | Y | Y | Y | N | NA | Y | Y | Y | Y | NA | Y | Low |
| Shigemi, 2023 | N | Y | Y | Y | N | Y | Y | N | Y | NA | Y | Moderate |
| Evangelopoulos, 2024 | Y | Y | Y | N | NA | Y | Y | N | Y | NA | Y | Moderate |
| Dehan, 2024 | N | Y | Y | Y | N | Y | Y | N | Y | NA | Y | Moderate |
| Billone, 2024 | N | Y | Y | N | NA | Y | Y | Y | NA | NA | Y | Moderate |
| Zhang, 2025 | Y | Y | Y | N | NA | Y | Y | Y | Y | NA | Y | Low |

**Figure S1.** Results of the random-effect meta-analysis for operative times in RCTs with the exclusion of Nilsson et al. (2024)


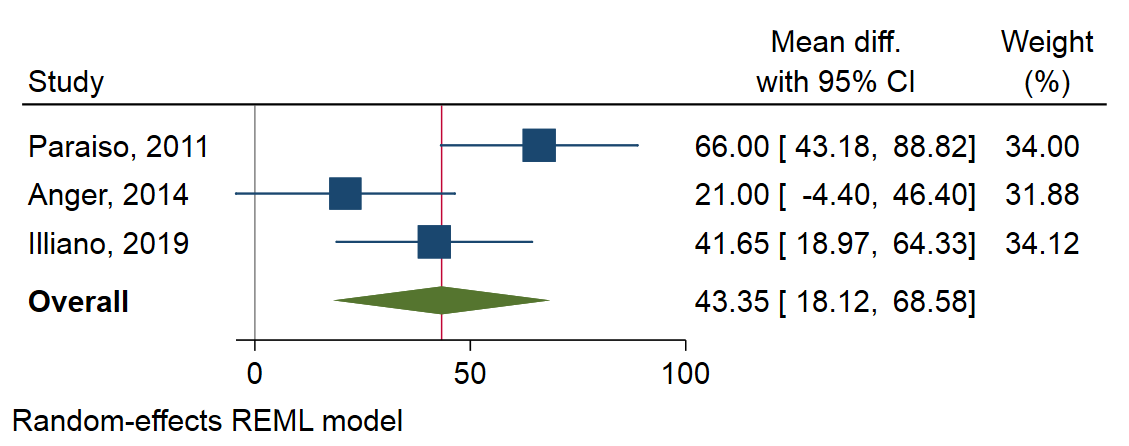


**Figure S2.** Results of the random-effect meta-analysis for complications in RCTs

**
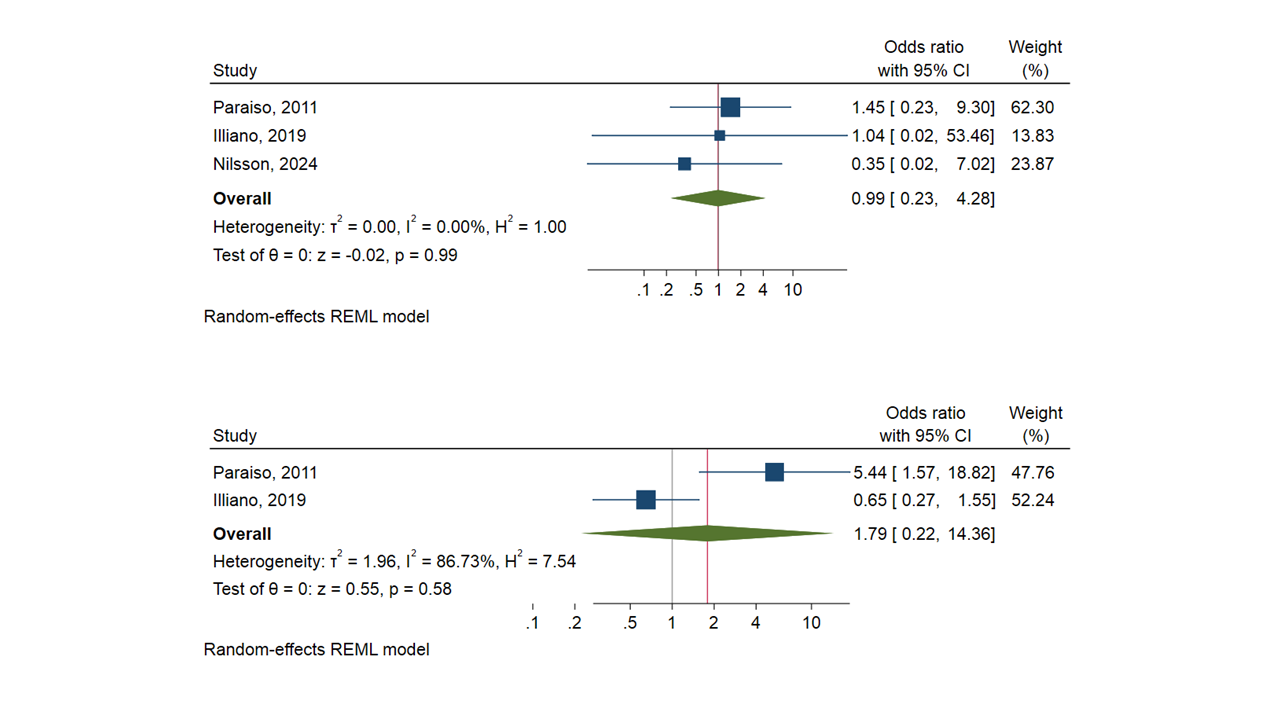
**

**Figure S3.** Funnel plots assessing publication bias for each outcome in observational studies


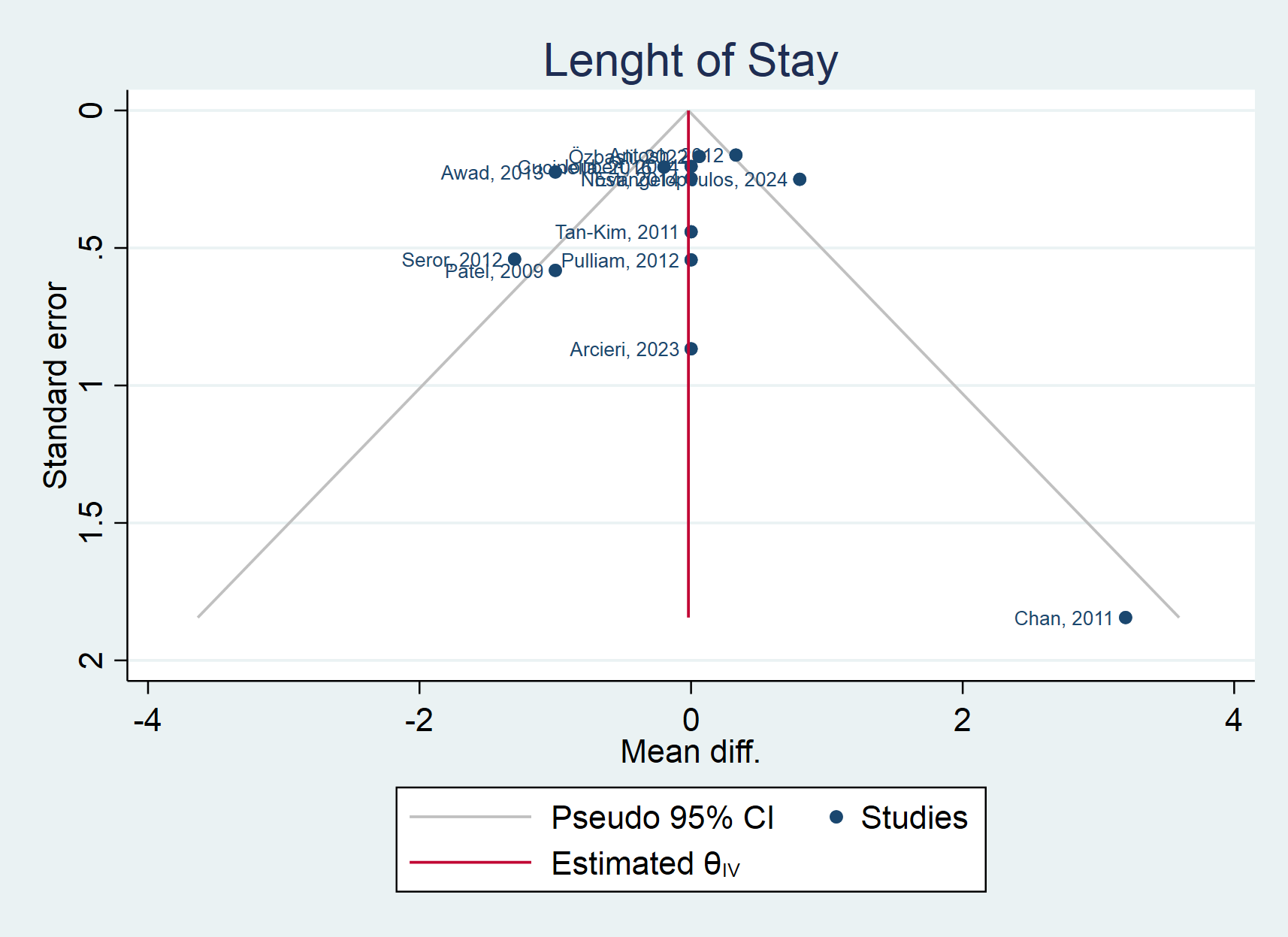

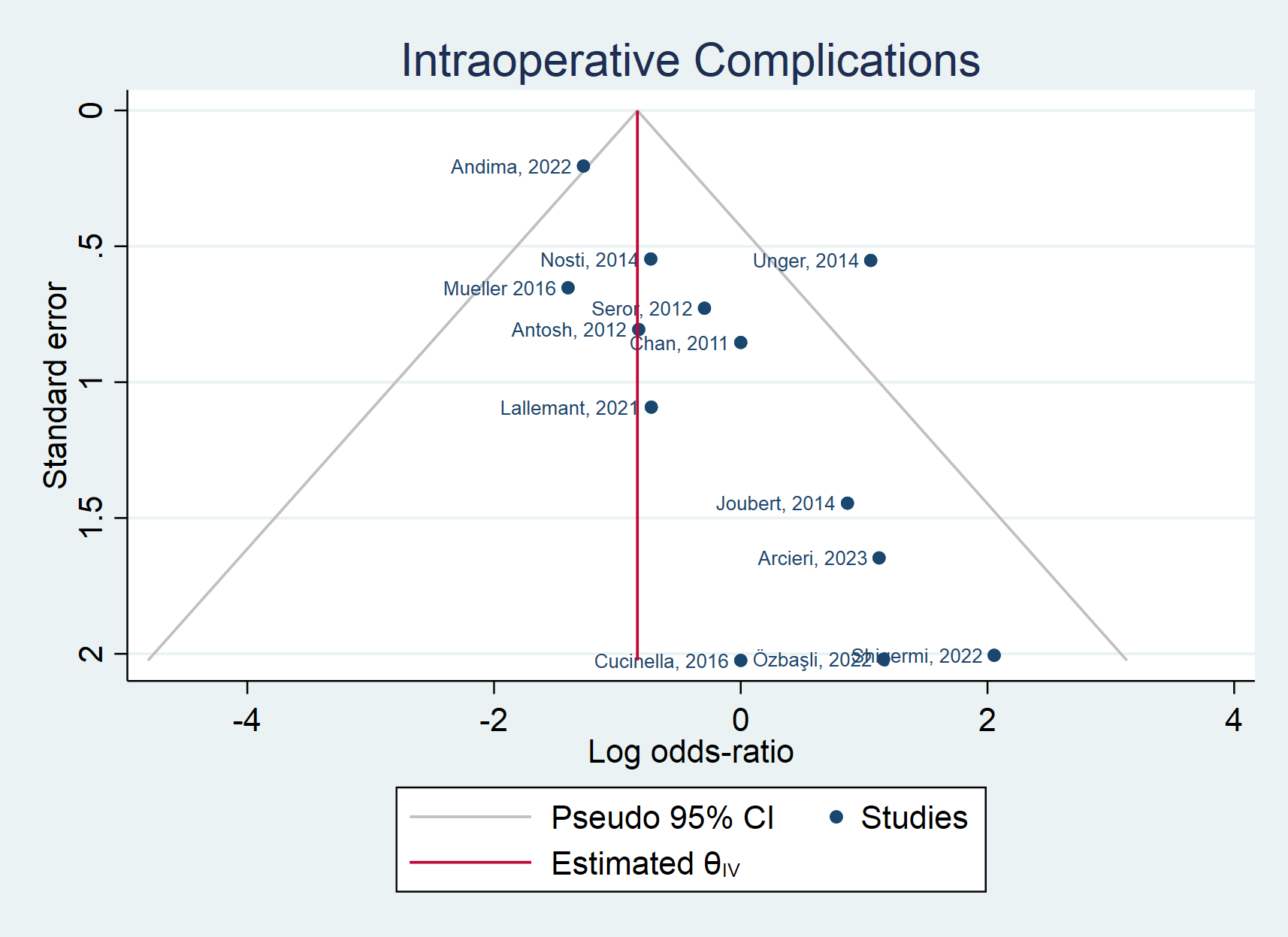


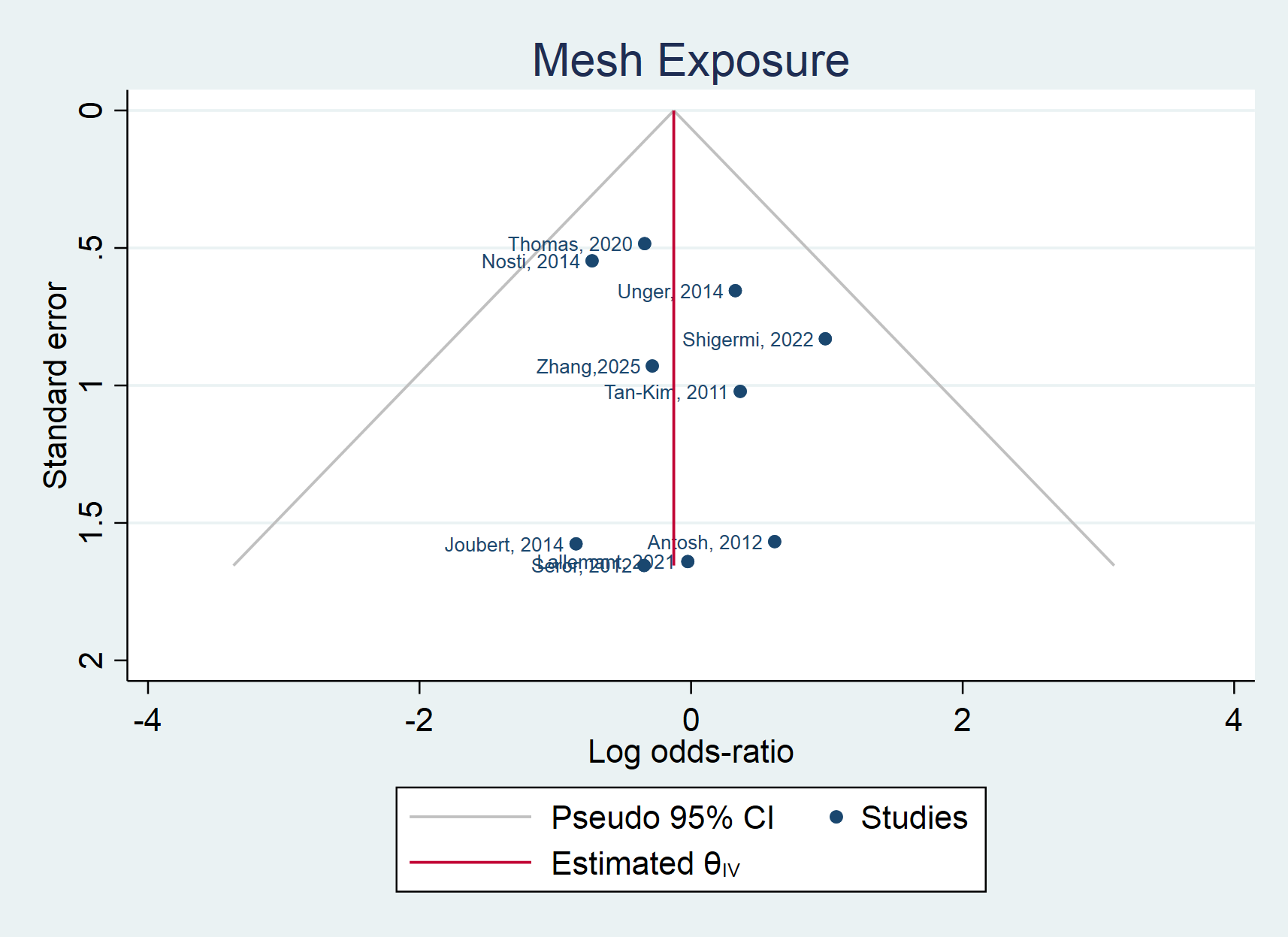

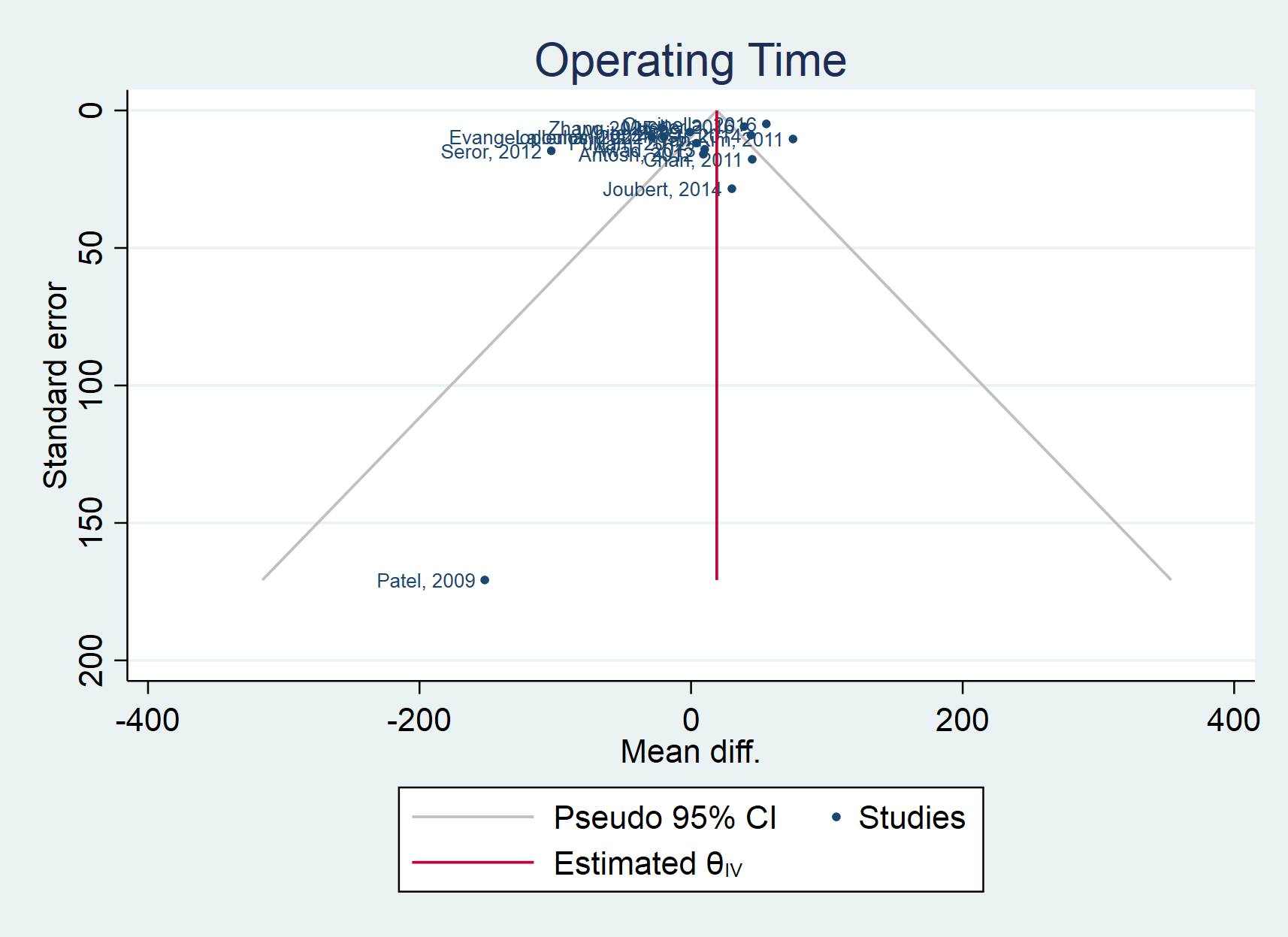

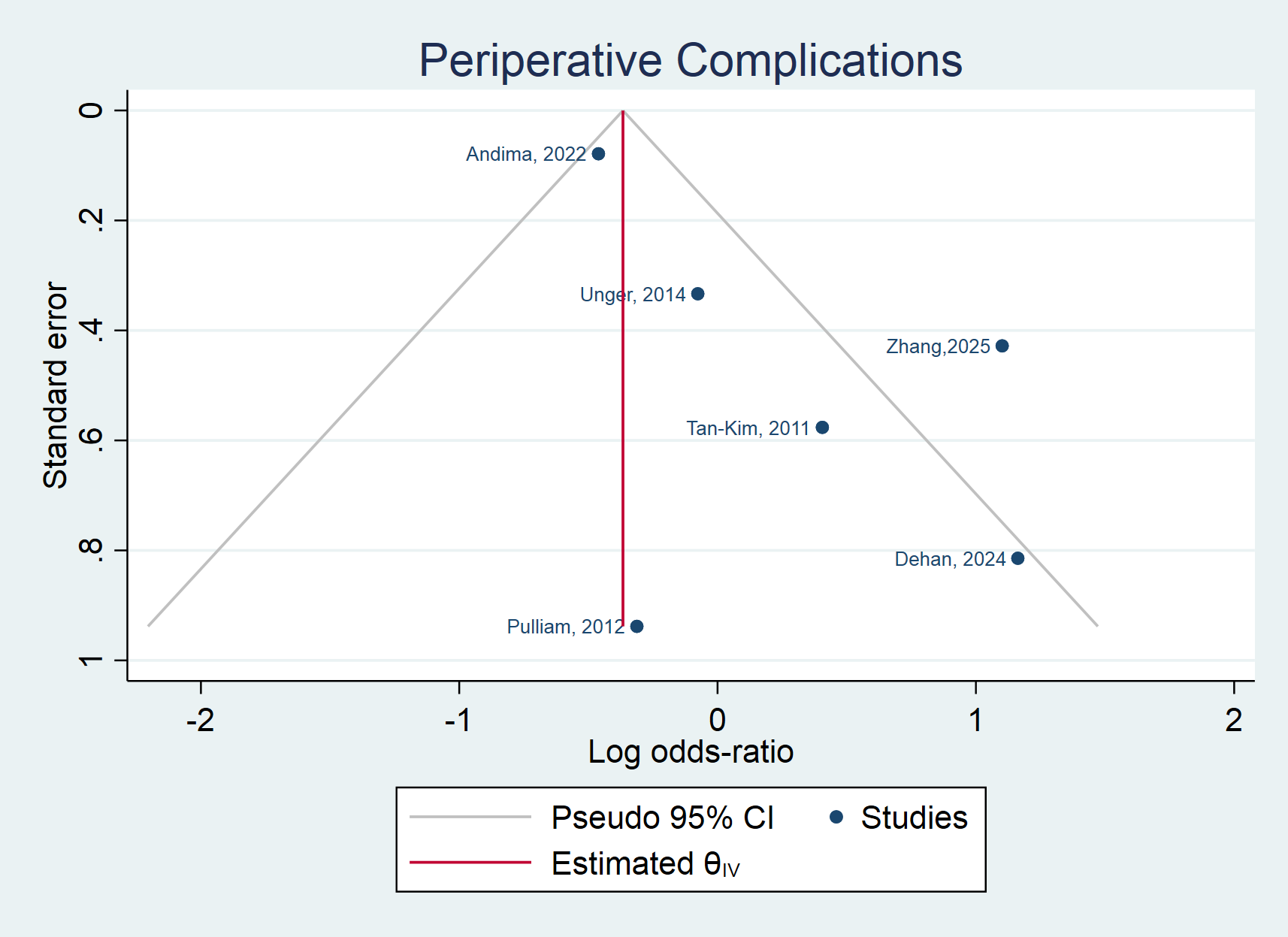

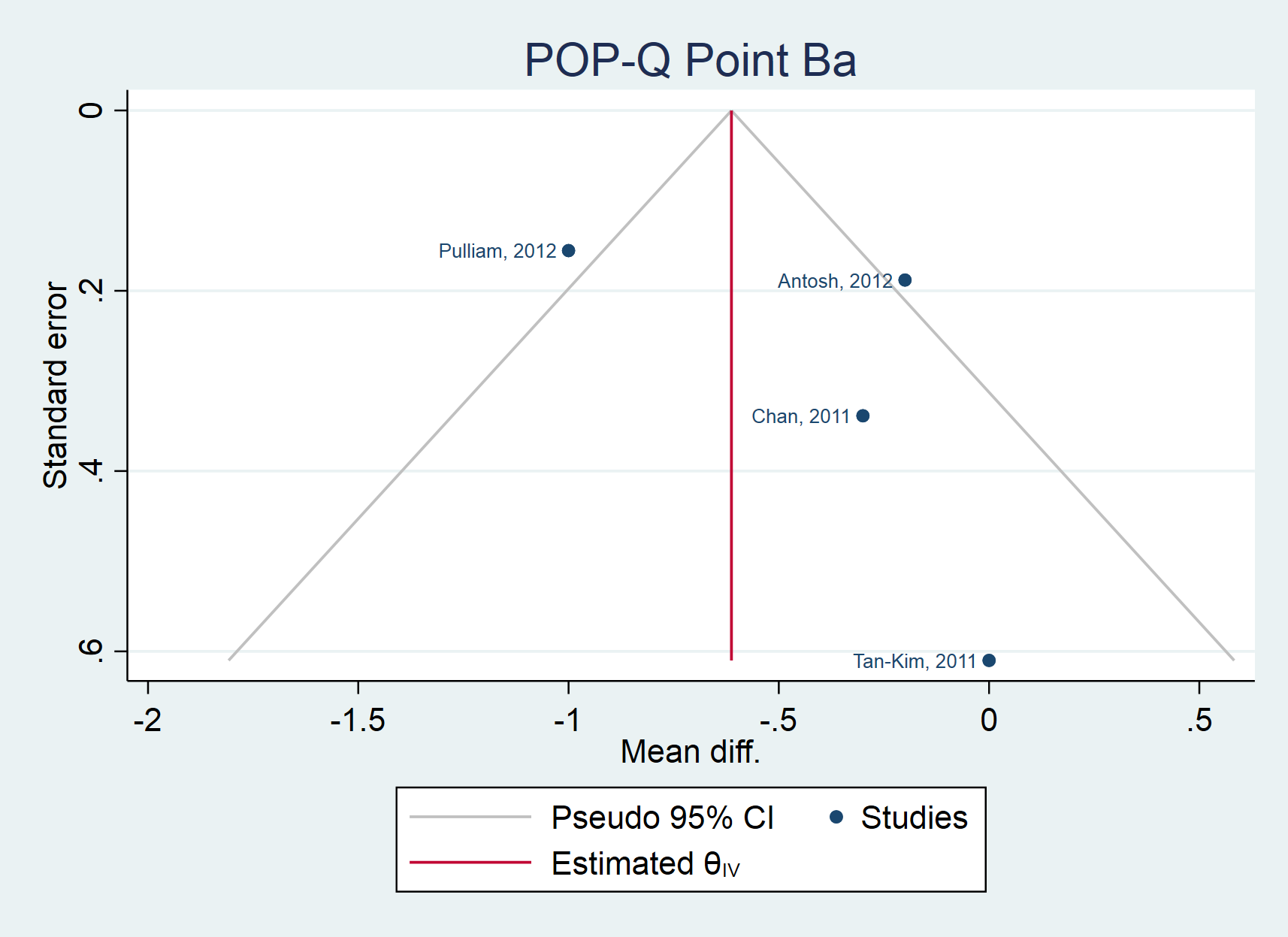

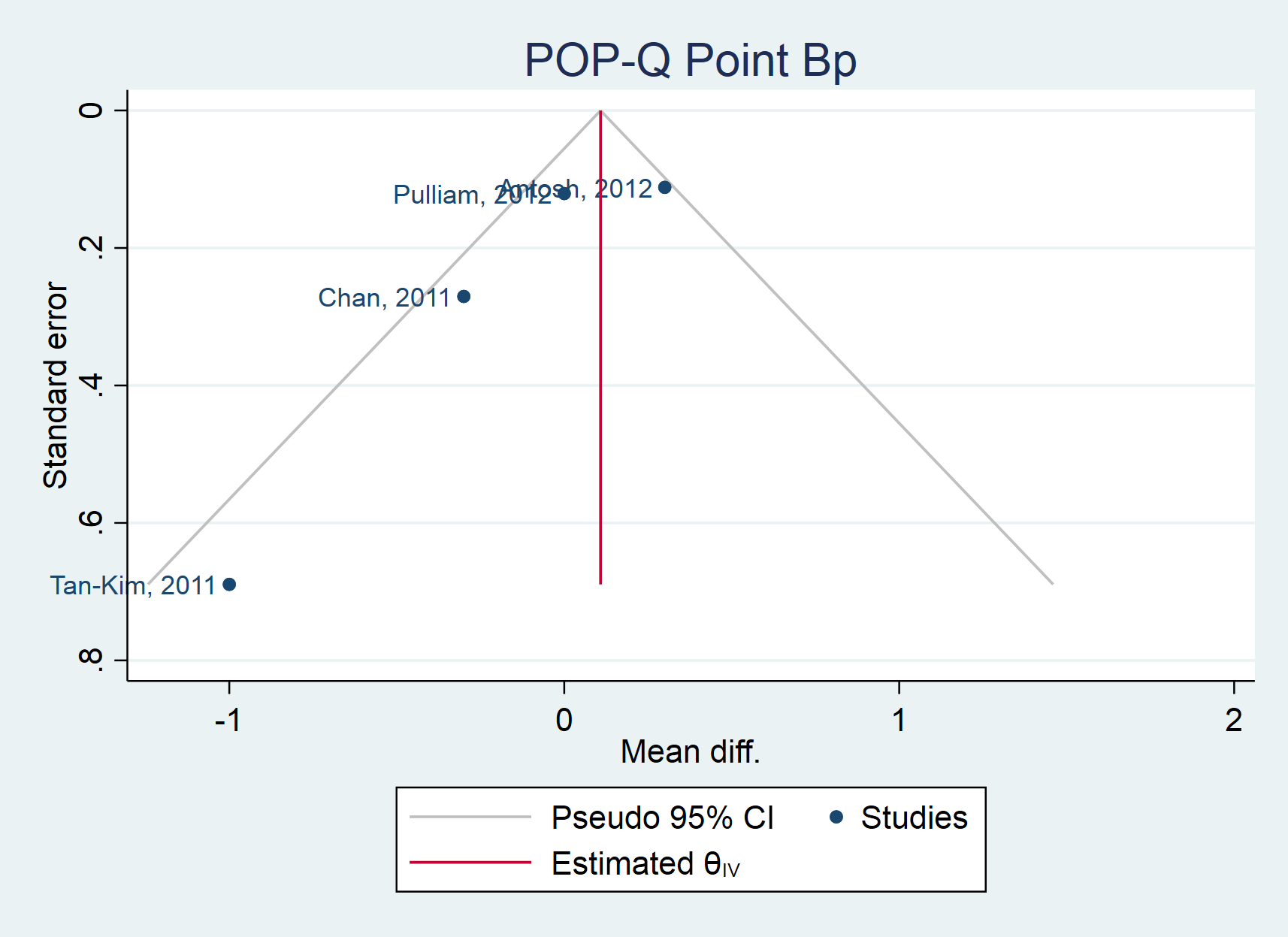

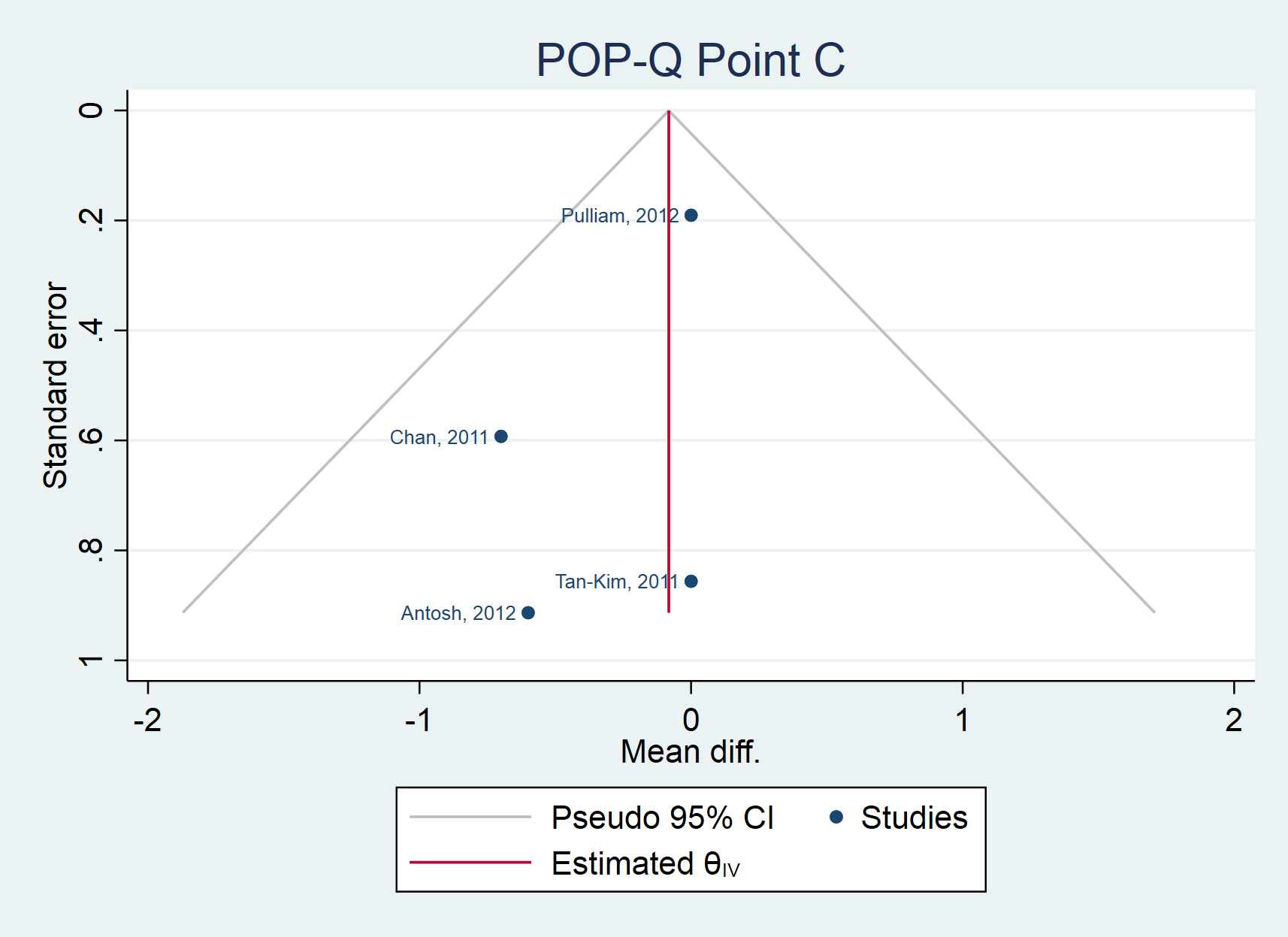

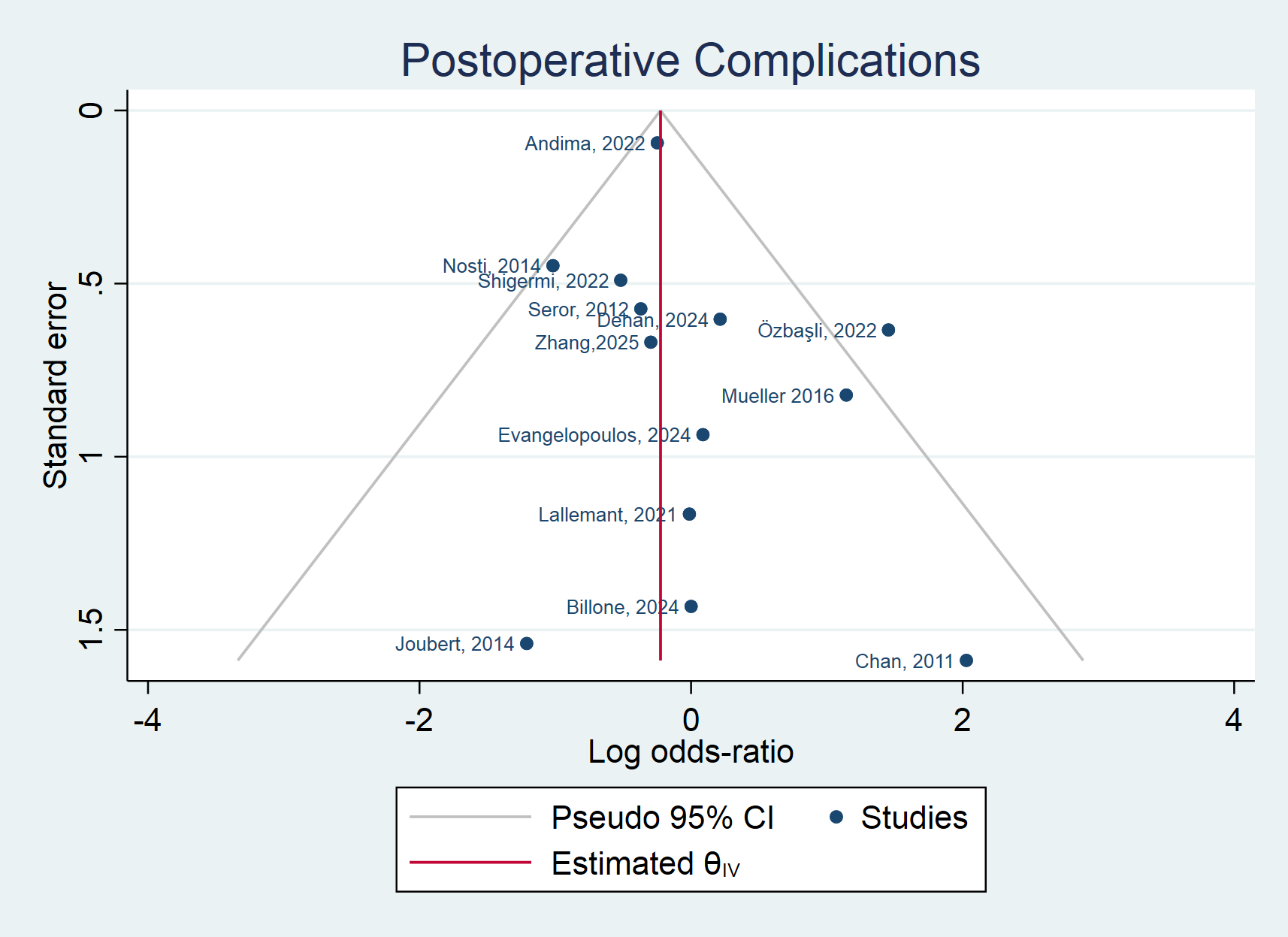

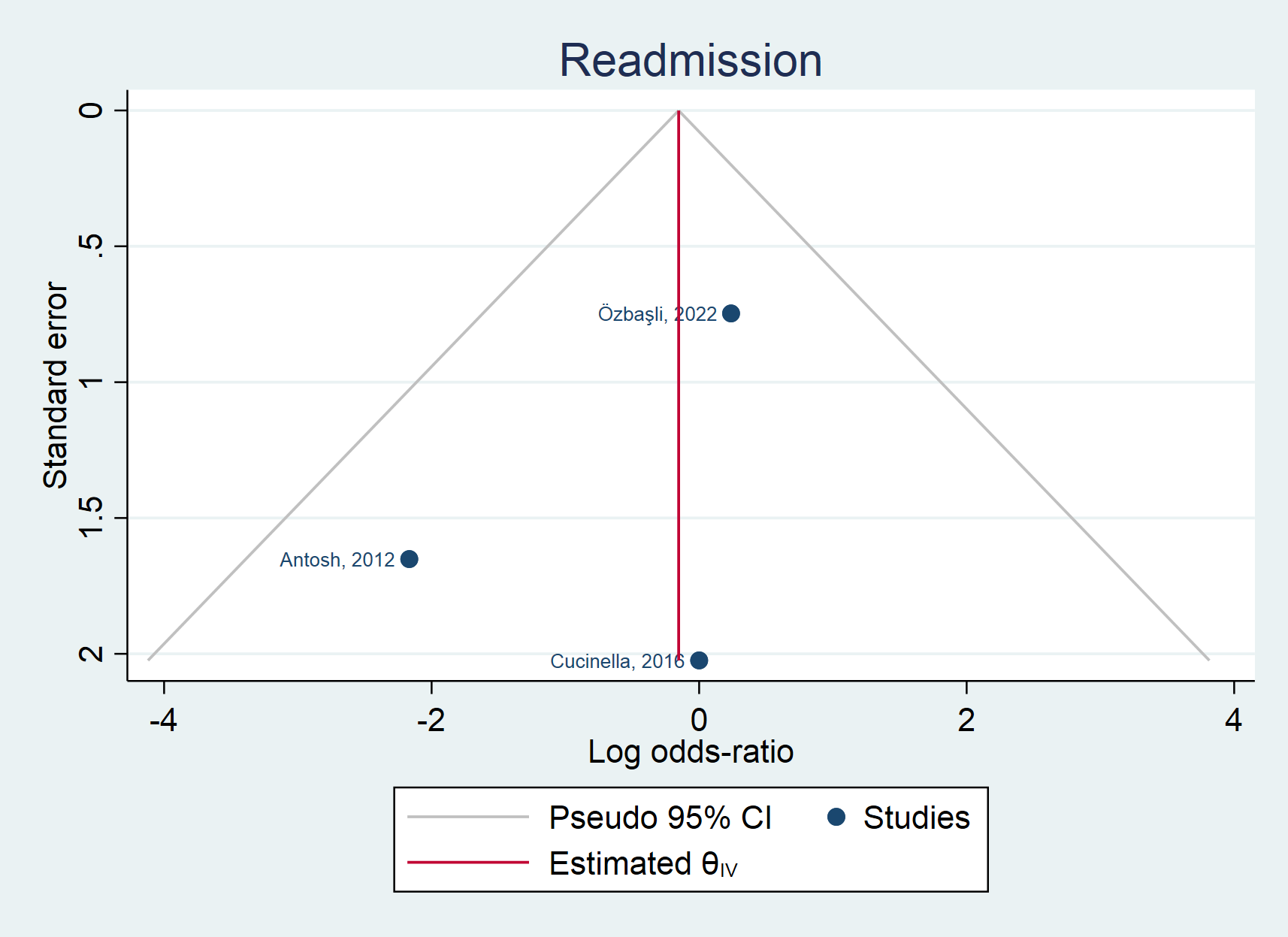

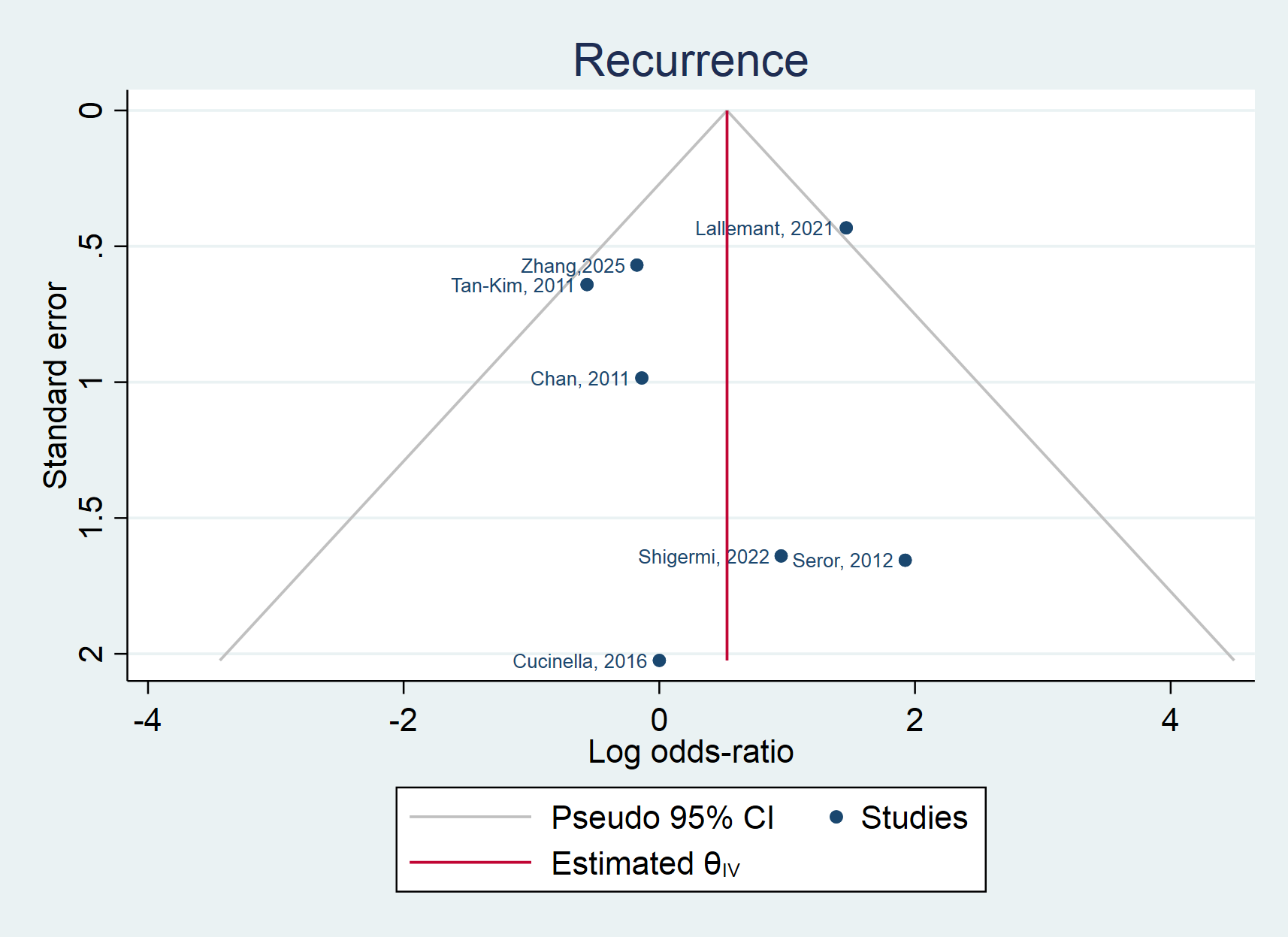

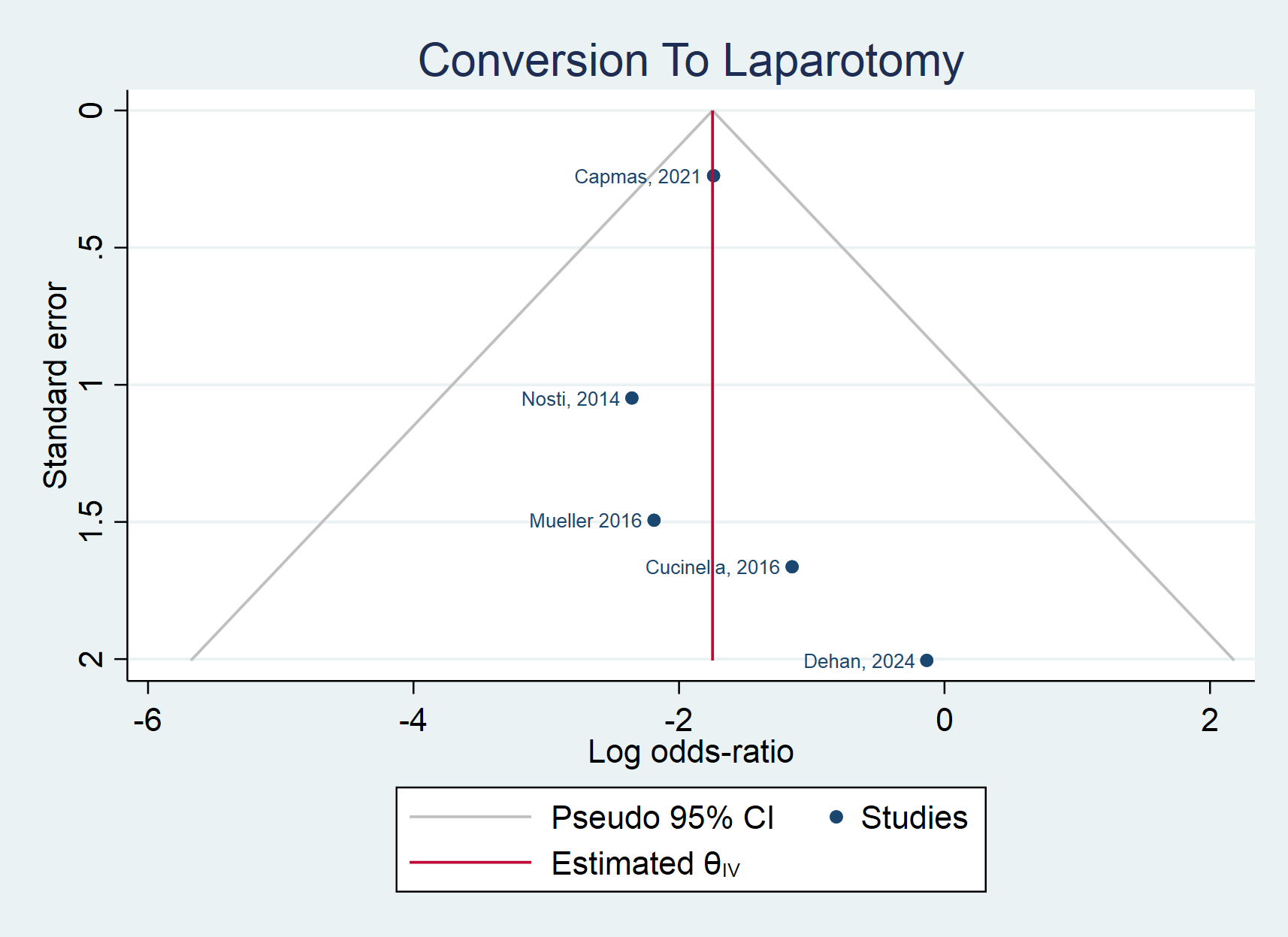

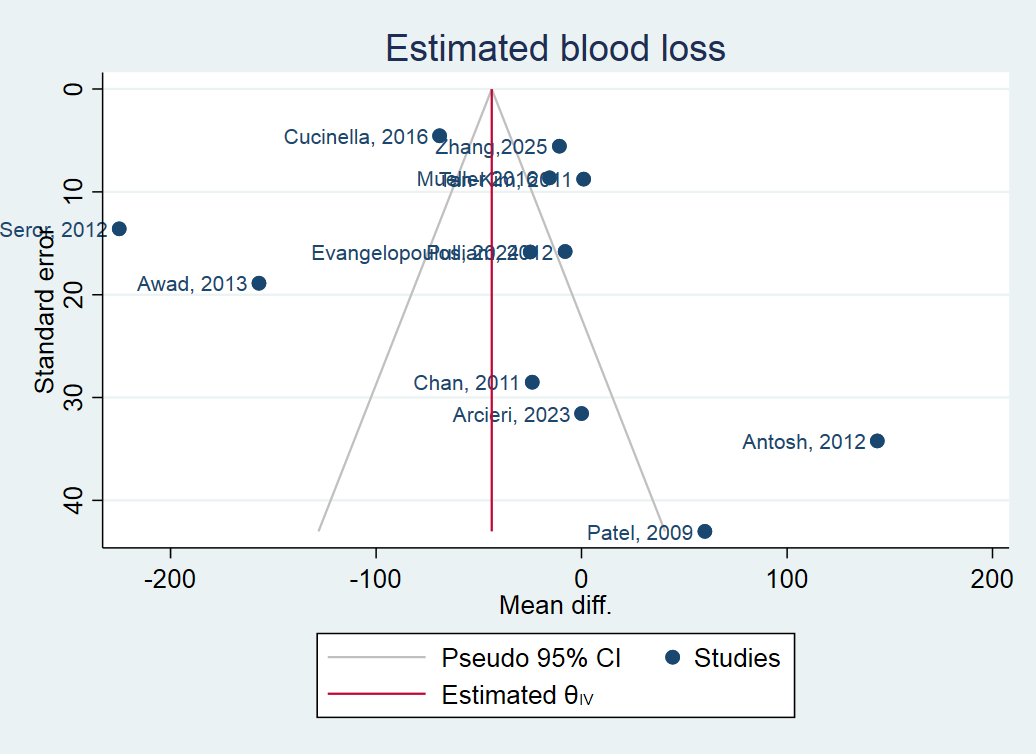

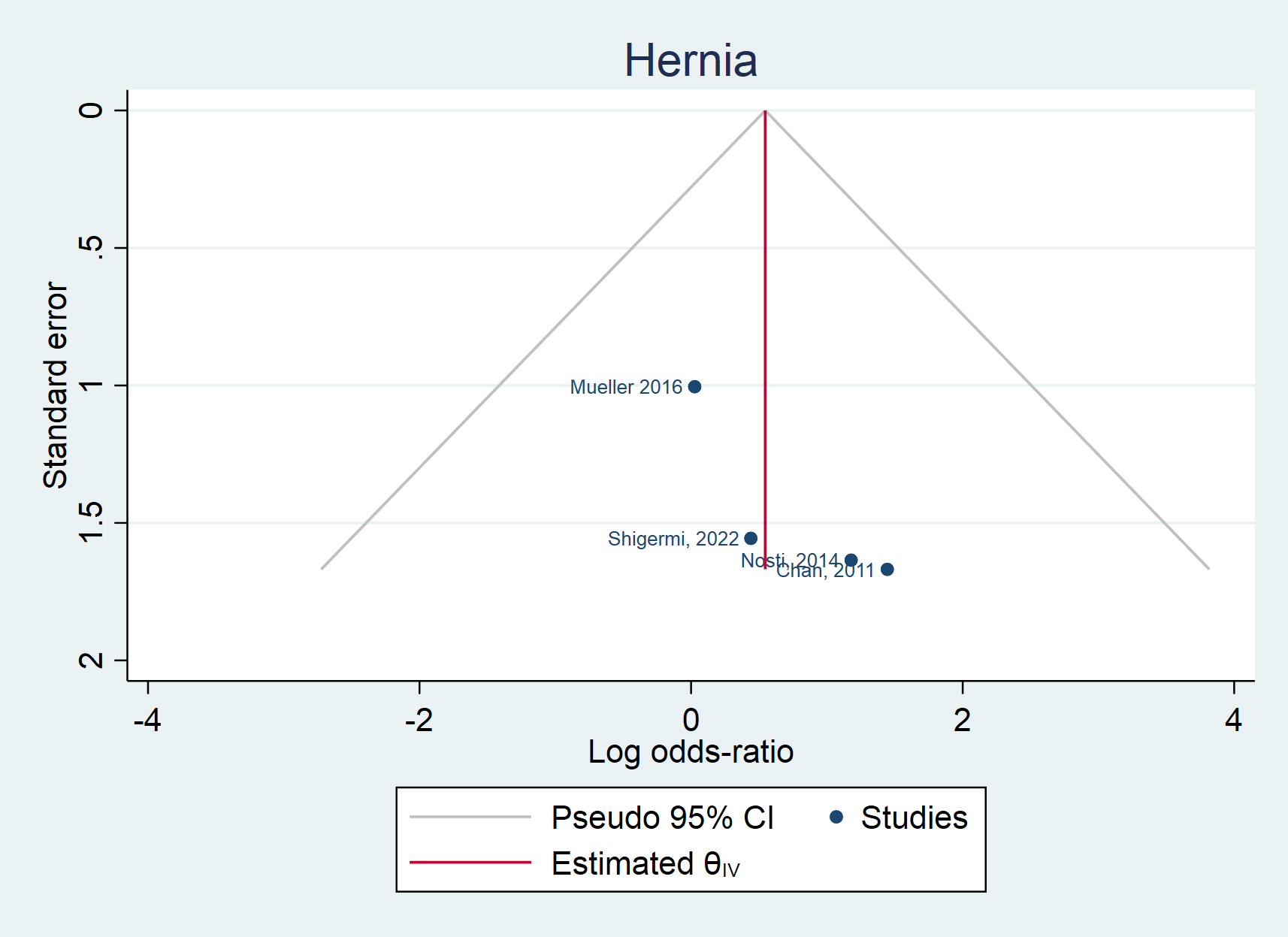

Supplement: Supplementary file 1 — Table S1: Search strategy for databases. Table S2: Characteristics of studies included in systematic review and meta‐analysis. Table S3: Study Quality Assessment for Observational studies. Figure S1: Results of the random‐effect meta‐analysis for operative times in RCTs with the exclusion of Nilsson et al. Figure S2: Results of the random‐effect meta‐analysis for complications in RCTs. Figure S3:. Funnel plots assessing publication bias for each outcome in observational studies. [file BJO-133-1529-s001.docx]
